# Electrification in granular gases leads to constrained fractal growth

**DOI:** 10.1038/s41598-019-45447-x

**Published:** 2019-06-21

**Authors:** Chamkor Singh, Marco G. Mazza

**Affiliations:** 1 Max Planck Institute for Dynamics and Self-Organization (MPIDS), 37077 Göttingen, Germany; 2 Georg-August-Universität Göttingen, Friedrich-Hund-Platz 1, 37077 Göttingen, Germany; 3 Interdisciplinary Centre for Mathematical Modelling and Department of Mathematical Sciences, Loughborough University, Loughborough, Leicestershire LE11 3TU United Kingdom

**Keywords:** Statistical physics, Phase transitions and critical phenomena

## Abstract

The empirical observation of aggregation of dielectric particles under the influence of electrostatic forces lies at the origin of the theory of electricity. The growth of clusters formed of small grains underpins a range of phenomena from the early stages of planetesimal formation to aerosols. However, the collective effects of Coulomb forces on the nonequilibrium dynamics and aggregation process in a granular gas – a model representative of the above physical processes – have so far evaded theoretical scrutiny. Here, we establish a hydrodynamic description of aggregating granular gases that exchange charges upon collisions and interact via the long-ranged Coulomb forces. We analytically derive the governing equations for the evolution of granular temperature, charge variance, and number density for homogeneous and quasi-monodisperse aggregation. We find that, once the aggregates are formed, the granular temperature of the cluster population, the charge variance of the cluster population and the number density of the cluster population evolve in such a way that their non-dimensional combination obeys a physical constraint of nearly constant dimensionless ratio of characteristic electrostatic to kinetic energy. This constraint on the collective evolution of charged clusters is confirmed both by  our theory and our detailed molecular dynamics simulations. The inhomogeneous aggregation of monomers and clusters in their mutual electrostatic field proceeds in a fractal manner. Our theoretical framework is extendable to more precise charge exchange mechanisms, a current focus of extensive experimentation. Furthermore, it illustrates the collective role of long-ranged interactions in dissipative gases and can lead to novel designing principles in particulate systems.

## Introduction

The electrostatic aggregation of small particles is ubiquitous in nature and ranks among the oldest scientific observations. Caused by collisional or frictional interactions among grains, large amounts of positive and negative charges can be generated. These clusters have far-reaching consequences: from aerosol formation to nanoparticle stabilization^1,2^, planetesimal formation, and the dynamics of the interstellar dust^3–6^. The processes accompanying granular collisions, charge buildup and subsequent charge separation can also lead to catastrophic events such as silo failure, or dust explosions.

Experimental investigations of the effects of tribocharging date back to Faraday, and recent *in situ* investigations have revealed important results^7–11^. However, technical difficulties plague even careful experiments and often impede their unambiguous interpretation^12^. A source of these difficulties is the lack of consensus about whether electrostatics facilitate or hinder the aggregation process of a large collection of granular particles^12^. Despite considerable effort^13–24^ a statistico-mechanical description of aggregation in a dissipative granular system with a mechanism of charge transfer is still lacking. The theoretical treatment requires reconsideration of the dissipation of kinetic energy conventionally described by a monotonic dependence of the coefficient of restitution on collision velocities $\epsilon (v)$, and also inclusion of long-range electrostatic forces due to the dynamically-changing charge production. Understanding the growth of charged aggregates requires a statistical approach due to the different kinetic properties and aggregate morphology.

In this work, we present a modified Boltzmann description for the inelastic and aggregative collisions of grains that interact via Coulomb forces, and exchange charges upon collision. We derive the hydrodynamic equations for the number density *n*, the granular temperature *T*, and the charge fluctuations $\langle \delta {q}^{2}\rangle$ of the aggregates under the assumptions of homogeneous and quasi-monodisperse aggregation. We find that the dimensionless ratio of the characteristic electrostatic energy ${k}_{e}\langle \delta {q}^{2}\rangle /d$ to the granular temperature *T*, *i*.*e*. ${k}_{e}\langle \delta {q}^{2}\rangle /(Td)$, approaches but stays below  unity. Here ${k}_{e}$ is the Coulomb’s constant while *d* is the characteristic size of the aggregates.

To bolster our results, and explicitly consider fluctuations in dynamics and morphological structures, we also use three-dimensional molecular dynamics (MD) simulations that explicitly include Coulombic interactions and a charge-exchange mechanism. We find that the granular dynamics agree quantitatively with the predictions of the Boltzmann equation. The cooling gas undergoes a transition from a dissipative to an aggregative phase marked by a crossover in the advective transport. We explore the morphological dynamics of the inhomogeneous aggregation via the mean fractal dimension and their interplay with the mesoscopic flow.

## Kinetics

**Figure 1:**
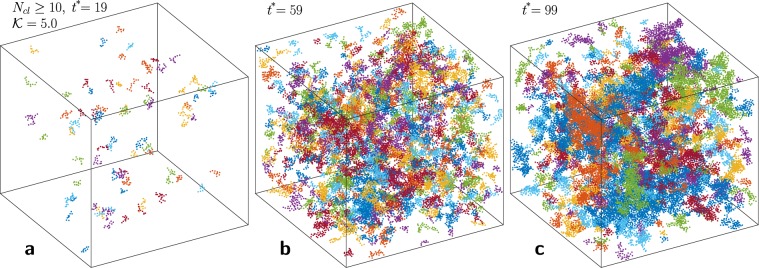
Snapshots of the aggregating charged granular gas at different non-dimensional times: (**a**) ${t}^{\ast }=19$, (**b**) ${t}^{\ast }=59$, and (**c**) ${t}^{\ast }=99$. Here clusters containing 10 particles or more (${N}_{cl}\ge 10$) are shown, and each color represents a different cluster. Clusters are identified on the basis of the monomer distances: if the centers of two particles are separated by a particle diameter, or less, they belong to the same cluster. See Methods for non-dimensional time ${t}^{\ast }=t{v}_{{\rm{ref}}}/{d}_{0}$, Coulomb force strength ${\mathscr{K}}$, and other reference scales.

In general, agglomeration in a three-dimensional collisionally charging cooling granular gas is a spatially inhomogeneous process which involves the interplay between dissipation, time-varying size distribution of aggregates, charge fluctuations and exchange mechanism during collisions, long-range forces, and collective effects^25^. This complexity is illustrated in Fig. 1 which shows snapshots of cooling clusters from a typical MD simulation, beginning from a homogeneous and neutral state (see Methods and^25^ for MD). In the following we establish a modified Boltzmann approach for this intricate dynamics of the aggregation process, which predicts novel physical limits.

**Figure 2:**
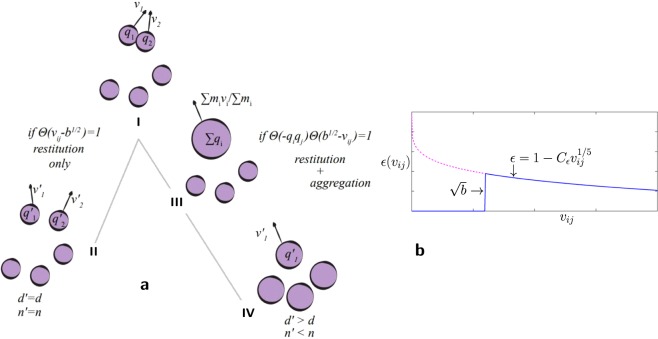
(**a**) When charged particles collide (particle group I), two possible pathways are considered in the kinetic theory depending on their velocities and charges: (*i*) a typical inelastic collision (dissipation with velocity dependent coefficient of restitution) with charge exchange (particle group II) if the relative collision speed is above a threshold speed $\sqrt{b}$, and (*ii*) a collision that leads to the aggregation (with coefficient of restitution equal to zero) and merging of charges (particle group III), if the relative collision speed is below $\sqrt{b}$ and the particles are oppositely charged. The latter event introduces a size distribution of aggregates, which we further simplify in our kinetic theory by adjusting the size of all the particles (particle group IV). Thus, the particle size is assumed to remain monodispersed during the course of aggregation. Primed variables represent post-collision or post-aggregation values. (**b**) Coefficient of restitution $\epsilon ({v}_{ij})$ in the present kinetic theory. Notice that the threshold $\sqrt{b}=\sqrt{\frac{2{k}_{e}|{q}_{i}{q}_{j}|}{md}}$ also evolves with time due to charge-exchange and aggregation events, and the coefficient of restitution is zero only if ${\rm{\Theta }}(\,-\,{q}_{i}{q}_{j}){\rm{\Theta }}({b}^{1/2}-{v}_{ij})=1$. The above kinetic approach is compared with the MD simulations where the size distribution of aggregates is polydisperse.

We consider the single particle probability distribution function $f=f({\bf{r}},t;{\bf{v}},q,d)$, where the particle velocity **v**, charge *q*, position **r** and particle size *d*, are the phase space variables, and *t* denotes time. We specialize to a homogeneous and quasi-monodisperse aggregation scenario (*i*.*e*. the size is assumed to vary in time but spatially mono-dispersed, see schematic representation in Fig. 2). Under these limits, the spatial and particle-size dependence of *f* drops out, *i*.*e*. $f=f(t;{\bf{v}},q)$ only, and its time evolution is given by the simplified Boltzmann equation^26,27^

1 $$\frac{\partial f}{\partial t}={I}_{{\rm{coll}}},$$

valid at any time instant *t*. Here we define *I*_coll_ as the modified collision integral which includes dissipation as well as charge exchange during particle collisions. We will now elucidate how the charge exchange mechanism and particle size growth modify the collisions.

Let us consider contact collisions of particles *i* and *j* with pre-collision velocity-charge values $({{\bf{v}}}_{i},{q}_{i})$ and $({{\bf{v}}}_{j},{q}_{j})$, respectively. In the ensemble picture, particle collisions will change $f(t;{\bf{v}},q)$ in the infinitesimal phase-space volumes $d{{\bf{v}}}_{i}d{q}_{i}$ and $d{{\bf{v}}}_{j}d{q}_{j}$, centered around $({{\bf{v}}}_{i},{q}_{i})$ and $({{\bf{v}}}_{j},{q}_{j})$, respectively. The number of direct collisions ${N}_{c}^{-}$ per unit spatial volume which lead to loss of particles from the intervals $d{{\bf{v}}}_{i}d{q}_{i}$ and $d{{\bf{v}}}_{j}d{q}_{j}$ in time Δ*t* are

2 $${N}_{c}^{-}={f}_{i}d{{\bf{v}}}_{i}d{q}_{i}{f}_{j}d{{\bf{v}}}_{j}d{q}_{j}|{{\bf{v}}}_{ij}\cdot {\bf{n}}|{\rm{\Theta }}(\,-\,{{\bf{v}}}_{ij}\cdot {\bf{n}}){{\rm{\Theta }}}_{q}d\sigma {\rm{\Delta }}t,$$

where ${{\bf{v}}}_{ij}\equiv {{\bf{v}}}_{j}-{{\bf{v}}}_{i}$, **n** is the unit vector at collision pointing from the center of particle *i* towards particle *j*, and *dσ* is the differential collision cross-section [Eq. (38)]. The Heaviside step function ${\rm{\Theta }}(\,-\,{{\bf{v}}}_{ij}\cdot {\bf{n}})$ selects particles coming towards *i*, while we use ${{\rm{\Theta }}}_{q}\equiv {\rm{\Theta }}(\frac{1}{2}m{v}_{ij}^{2}-\frac{{k}_{e}{q}_{i}{q}_{j}}{d})$ to ensure that a contact with an approaching particle takes place only when the Coulomb energy barrier can be overcome, where ${k}_{e}=\mathrm{1/(4}\pi {\varepsilon }_{0})$, ${\varepsilon }_{0}=8.854\times {10}^{-12}\,{\rm{F}}\,{{\rm{m}}}^{-1}$ is the vacuum permittivity, and *d* is the particle diameter at time *t*. If the interaction is repulsive, ${k}_{e}{q}_{i}{q}_{j}/d$ is positive, and ${{\rm{\Theta }}}_{q}=1$ only if $\frac{1}{2}m{v}_{ij}^{2} > {k}_{e}{q}_{i}{q}_{j}/d$. In case of attractive interaction, ${k}_{e}{q}_{i}{q}_{j}/d$ is negative and thus ${{\rm{\Theta }}}_{q}=1$ always. Essentially, ${{\rm{\Theta }}}_{q}$ filters repulsive interactions which do not lead to a physical contact between particles.

Consider now particles with initial velocity-charge values $({{\bf{v}}}_{i}^{{\rm{^{\prime} }}{\rm{^{\prime} }}},{q}_{i}^{{\rm{^{\prime} }}{\rm{^{\prime} }}})$ and $({{\bf{v}}}_{j}^{{\rm{^{\prime} }}{\rm{^{\prime} }}},{q}_{j}^{{\rm{^{\prime} }}{\rm{^{\prime} }}})$ in the intervals ${d{\bf{v}}}_{i}^{{\rm{^{\prime} }}{\rm{^{\prime} }}}d{q}_{i}^{{\rm{^{\prime} }}{\rm{^{\prime} }}}$, ${d{\bf{v}}}_{j}^{{\rm{^{\prime} }}{\rm{^{\prime} }}}d{q}_{j}^{{\rm{^{\prime} }}{\rm{^{\prime} }}}$. The number of particles ${N}_{c}^{+}$  (inverse collisions) per unit volume which, post-collision, enter the interval $d{{\bf{v}}}_{i}d{q}_{i}$ and $d{{\bf{v}}}_{j}d{q}_{j}$ in time Δ*t* is

3 $${N}_{c}^{+}={f}_{i}^{{\rm{^{\prime} }}{\rm{^{\prime} }}}{d{\bf{v}}}_{i}^{{\rm{^{\prime} }}{\rm{^{\prime} }}}d{q}_{i}^{{\rm{^{\prime} }}{\rm{^{\prime} }}}{f}_{j}^{{\rm{^{\prime} }}{\rm{^{\prime} }}}{d{\bf{v}}}_{j}^{{\rm{^{\prime} }}{\rm{^{\prime} }}}d{q}_{j}^{{\rm{^{\prime} }}{\rm{^{\prime} }}}|{{\bf{v}}}_{ij}^{{\rm{^{\prime} }}{\rm{^{\prime} }}}\cdot {\bf{n}}|{\rm{\Theta }}(\,-\,{{\bf{v}}}_{ij}^{{\rm{^{\prime} }}{\rm{^{\prime} }}}\cdot {\bf{n}}){{\rm{\Theta }}}_{q}^{{\rm{^{\prime} }}{\rm{^{\prime} }}}d{\sigma }^{{\rm{^{\prime} }}{\rm{^{\prime} }}}{\rm{\Delta }}t.$$

The net change ${\rm{\Delta }}{N}_{c}\equiv {N}_{c}^{+}-{N}_{c}^{-}$ of number of particles in time Δ*t* per unit volume, then reads (see Methods)

4 $${\rm{\Delta }}{N}_{c}=(\frac{1}{\epsilon ({v}_{ij})}{J}_{ij}^{v}{J}_{ij}^{q}{f}_{i}^{{\rm{^{\prime} }}{\rm{^{\prime} }}}{f}_{j}^{{\rm{^{\prime} }}{\rm{^{\prime} }}}-{f}_{i}{f}_{j})|{{\bf{v}}}_{ij}\cdot {\bf{n}}|{\rm{\Theta }}(\,-\,{{\bf{v}}}_{ij}\cdot {\bf{n}})d{{\bf{v}}}_{j}d{q}_{j}d\sigma {{\rm{\Theta }}}_{q}{\rm{\Delta }}t,$$

where ${J}_{ij}^{v}$ and ${J}_{ij}^{q}$ are the Jacobians of the transformations for $d{{\bf{v}}}_{i}^{{\rm{^{\prime} }}{\rm{^{\prime} }}}d{{\bf{v}}}_{j}^{{\rm{^{\prime} }}{\rm{^{\prime} }}}\to d{{\bf{v}}}_{i}d{{\bf{v}}}_{j}$ and $d{q}_{i}^{{\rm{^{\prime} }}{\rm{^{\prime} }}}d{q}_{j}^{{\rm{^{\prime} }}{\rm{^{\prime} }}}\to d{q}_{i}d{q}_{j}$, respectively, which lump together the microscopic details of the collision process, namely dissipation and charge exchange in the present study.

Integrating over all incoming particle velocities and charges from all directions, dividing by Δ*t* and taking the limit ${\rm{\Delta }}t\to 0$, we obtain the formal expression for the modified collision integral

5 $${I}_{{\rm{c}}{\rm{o}}{\rm{l}}{\rm{l}}}=\int (\frac{1}{\epsilon ({v}_{ij})}{J}_{ij}^{v}{J}_{ij}^{q}{f}_{i}^{{\rm{^{\prime} }}{\rm{^{\prime} }}}{f}_{j}^{{\rm{^{\prime} }}{\rm{^{\prime} }}}-{f}_{i}{f}_{j})|{{\bf{v}}}_{ij}\cdot {\bf{n}}|{\rm{\Theta }}(\,-\,{{\bf{v}}}_{ij}\cdot {\bf{n}})d{{\bf{v}}}_{j}d{q}_{j}d\sigma {{\rm{\Theta }}}_{q}.$$

Here we assume that the differential collision cross section and the contact condition specified by ${{\rm{\Theta }}}_{q}$ retain their form for direct and inverse collisions. The particle encounters which do not lead to a physical contact have been excluded using ${{\rm{\Theta }}}_{q}$. While taking moments of *I*_coll_, a fraction of those contact collisions that lead to aggregation is accounted for by taking the limit $\epsilon =0$ for certain conditions on the relative velocity *v*_*ij*_, and by considering the charge transferred to particle *i* equal to the charge on particle *j*. In *I*_coll_, distant encounters, which do not lead to a contact between particles (glancing collisions) are neglected and the charge exchange and dissipation is considered only during the contact. The long-range effect is incorporated via the collision cross-section [Eq. (38)].

After setting up the collision integral, we derive the macroscopic changes of number density $n=\frac{{N}_{{\rm{agg}}}}{V}$, granular temperature $\frac{3}{2}T=\langle \frac{1}{2}{m}_{k}{{\bf{v}}}_{k}^{2}\rangle$, and the charge variance $\langle \delta {q}^{2}\rangle =\langle {({q}_{k}-{q}_{{\rm{mean}}})}^{2}\rangle$ for a homogeneously aggregating granular gas by taking the moments of the Boltzmann equation (see Methods), in the absence of any macroscopic velocity **V**. Here *N*_agg_  is  the number of aggregates in a spatial volume *V*; ${m}_{k}$, ${{\bf{v}}}_{k}$ and *q*_*k*_ are the aggregate mass, velocity and charge respectively, while subscript “mean” signifies the mean value of the quantity. The particles are initially neutral and the charge on them is altered either by collisions or during aggregation. However, due to charge conservation during collisions and aggregation, the system remains globally neutral and the mean charge variation $\langle \delta q\rangle =\langle {q}_{k}-{q}_{{\rm{mean}}}\rangle$ is zero. The next choice is thus $\langle \delta {q}^{2}\rangle$. In order to obtain closed form equations, and for analytical tractability, we assume quasi-monodispersity and homogeneity of the aggregating granular gas at any given time, as illustrated in Fig. 2. This means that during aggregation the mass of the clusters is assumed to grow homogeneously, while their numbers decrease in a given volume.

We assume that the charge and velocity distributions are uncorrelated, and their properly scaled form remains Gaussian. After integration we find the governing equations

6 $$\frac{\partial n}{\partial t}=-\,{n}^{2}{T}^{\frac{1}{2}}{g}_{1}( {\mathcal B} ,{C}_{agg}^{n}),$$

7 $$\frac{3}{2}\frac{\partial T}{\partial t}=-\,{n}^{2}{T}^{\frac{8}{5}}{g}_{2}( {\mathcal B} ,{C}_{res}^{T})+{n}^{2}{T}^{\frac{3}{2}}{g}_{3}( {\mathcal B} ,{C}_{agg}^{T}),$$

8 $$\frac{\partial \langle \delta {q}^{2}\rangle }{\partial t}={n}^{2}{T}^{(\eta +\frac{1}{2})}{g}_{4}( {\mathcal B} ,{C}_{res}^{q})-{n}^{2}\langle \delta {q}^{2}\rangle {T}^{\frac{1}{2}}{g}_{5}( {\mathcal B} ,{C}_{agg}^{q}),$$

which are coupled via a time-dependent dimensionless ratio

9 $$ {\mathcal B} (t)\equiv {k}_{e}\frac{\langle \delta {q}^{2}\rangle (t)}{T(t)d(t)},$$

between charge variance, granular temperature, and aggregate size. The terms *g*_*k*_ are time-dependent functions of ${\mathcal B}$ and material constants ${C}_{res}$, ${C}_{agg}$ (Methods Table 1 and Eq. (32–36)). We term the ratio ${\mathcal B}$ as Bjerrum number. In Eq. (9), *d* represents the size of a particle, also evolving with time during aggregation [Fig. 2]. Notice that as *f* is considered independent of *d* during aggregation, an explicit equation for *d* is required. For this we consider the total mass *M*, system volume *V*, and particle material density ${\rho }_{p}$ to be constant, which fixes the relation between particle size *d* and particle number density *n*, according to

10 $$d(t)={[\frac{6M}{\pi n(t)V{\rho }_{p}}]}^{\frac{1}{{D}_{f}}},$$

and closes the Eqs set (6–8). Here *D*_*f*_ is the fractal dimension. In the solution of the above analytical equations, we have assumed ${D}_{f}=3$ (spherical aggregates). Below, we will compute values of *D*_*f*_ using the MD simulations. The above set of equations is consistent with modified Haff’s law for a velocity dependent coefficient of restitution^28–30^ in the absence of collisional charging. In this limit $\langle \delta {q}^{2}\rangle =0$, ${\mathcal B} =0$, and we obtain $\frac{\partial n}{\partial t}=0$, $\frac{\partial \langle \delta {q}^{2}\rangle }{\partial t}=0$ and $\frac{3}{2}\frac{\partial T}{\partial t}=-\,{T}^{8/5}[\frac{\pi {C}_{res}^{T}}{2}]$, whose solution is the modified Haff’s law.

**Table 1:** Expressions of the coefficients in Eqs (5–7) in the main text.

Coefficient	Expression
${\mathcal B}$	${k}_{e}\langle \delta {q}^{2}\rangle /(Td)$
$l$	$\sqrt{4-{ {\mathcal B} }^{2}}$
${l}_{1}$	$\sqrt{1-{ {\mathcal B} }^{2}}$
${C}_{res}^{T}$	$80\,{2}^{1/5}\,{d}^{2}{C}_{\epsilon }m/(21\sqrt{\pi }{m}^{8/5})$
${C}_{agg}^{T}$	${d}^{2}\mathrm{/(8}\sqrt{\pi m})$
${C}_{res}^{q}$	$1.4080\,{d}^{2}{C}_{{\rm{\Delta }}q}^{2}\mathrm{(2}+\eta \mathrm{)(4/}m{)}^{\eta +\mathrm{1/2}}/\pi$
${C}_{agg}^{q}$	$4{d}^{2}/\sqrt{\pi m}$
${C}_{agg}^{n}$	${d}^{2}\mathrm{/(2}\sqrt{\pi m})$
${a}_{1}^{T}$	${\mathcal B} \mathrm{(8}-5{ {\mathcal B} }^{2})l+\pi \mathrm{(16}-10{ {\mathcal B} }^{2}+3{ {\mathcal B} }^{4})$
${a}_{2}^{T}$	$-\,32+20{ {\mathcal B} }^{2}-6{ {\mathcal B} }^{4}$
${a}_{3}^{T}$	$l\mathrm{(2}\pi {({ {\mathcal B} }^{2}-\mathrm{4)}}^{2}+ {\mathcal B} (\,-\,32+28{ {\mathcal B} }^{2}+{ {\mathcal B} }^{4}))$
${a}_{4}^{T}$	$-\,\mathrm{4(32}-20{ {\mathcal B} }^{2}+9{ {\mathcal B} }^{4})$
${a}_{5}^{T}$	$l {\mathcal B} (\,-\,32+28{ {\mathcal B} }^{2}+{ {\mathcal B} }^{4})$
	$$+\,2\pi ({ {\mathcal B} }^{2}\mathrm{(20}-8l)+{ {\mathcal B} }^{4}(\,-\,9+l)+\mathrm{16(}\,-\,2+l))$$
${a}_{6}^{T}$	$\mathrm{4(32}-20{ {\mathcal B} }^{2}+9{ {\mathcal B} }^{4})$
${a}_{1}^{q}$	$2 {\mathcal B} l({ {\mathcal B} }^{2}-\mathrm{1)}-\pi \mathrm{(4}-2{ {\mathcal B} }^{2}+{ {\mathcal B} }^{4})$
${a}_{2}^{q}$	$\mathrm{2(4}-2{ {\mathcal B} }^{2}+{ {\mathcal B} }^{4})$
${a}_{3}^{q}$	$16l\sqrt{\pi }{l}_{1}{\mathrm{(4}-5{ {\mathcal B} }^{2}+{ {\mathcal B} }^{4})}^{2}$
${a}_{4}^{q}$	$-\,\sqrt{\pi } {\mathcal B} l(\pi ({ {\mathcal B} }^{2}-\mathrm{4)(1}+2{ {\mathcal B} }^{2})$
	$$+\,2 {\mathcal B} {l}_{1}(\,-\,8-54{ {\mathcal B} }^{2}+33{ {\mathcal B} }^{4}+2{ {\mathcal B} }^{6}))$$
${a}_{5}^{q}$	$2\sqrt{\pi } {\mathcal B} {l}^{5}\mathrm{(1}+2{ {\mathcal B} }^{2})$
${a}_{6}^{q}$	$16\sqrt{\pi } {\mathcal B} {l}_{1}^{5}\mathrm{(4}+5{ {\mathcal B} }^{2})$
${a}_{7}^{q}$	$8l\sqrt{\pi }{l}_{1}{\mathrm{(4}-5{ {\mathcal B} }^{2}+{ {\mathcal B} }^{4})}^{2}$
${a}_{8}^{q}$	$-\,\sqrt{\pi } {\mathcal B} {l}^{5}\mathrm{(1}+2{ {\mathcal B} }^{2})$
${a}_{9}^{q}$	$\sqrt{\pi } {\mathcal B} {l}_{1} {\mathcal B} l\mathrm{(8}+54{ {\mathcal B} }^{2}-33{ {\mathcal B} }^{4}-2{ {\mathcal B} }^{6})$
${a}_{10}^{q}$	$8\sqrt{\pi } {\mathcal B} {l}_{1}{({ {\mathcal B} }^{2}-\mathrm{1)}}^{2}\mathrm{(4}+5{ {\mathcal B} }^{2})$
${a}_{1}^{n}$	$-\,{ {\mathcal B} }^{2}{l}^{3}$
${a}_{2}^{n}$	${l}_{1}l {\mathcal B} (\,-\,4+7{ {\mathcal B} }^{2})+{l}_{1}\pi ({ {\mathcal B} }^{2}-1)(8-4l+{ {\mathcal B} }^{2}(\,-\,6+l))$
${a}_{3}^{n}$	$4{l}_{1}\mathrm{(4}-7{ {\mathcal B} }^{2}+3{ {\mathcal B} }^{4})$
${a}_{4}^{n}$	$-\,{ {\mathcal B} }^{2}{l}^{3}$
${a}_{5}^{n}$	${l}_{1}l( {\mathcal B} (\,-\,4+7{ {\mathcal B} }^{2})+\pi \mathrm{(4}-5{ {\mathcal B} }^{2}+{ {\mathcal B} }^{4}))$
${a}_{6}^{n}$	$-\,4{l}_{1}(4-7{ {\mathcal B} }^{2}+3{ {\mathcal B} }^{4})$

Here $m$ and $d$ are the mass and size of the aggregates. The material constant ${C}_{\epsilon }$ [Eq. (31)] influences the viscoelastic properties of the particles, while ${C}_{{\rm{\Delta }}q}$ and $\eta$ [Eq. (28)] influence the charge buildup. Other notations are as described in the main text.

## Results

**Figure 3:**
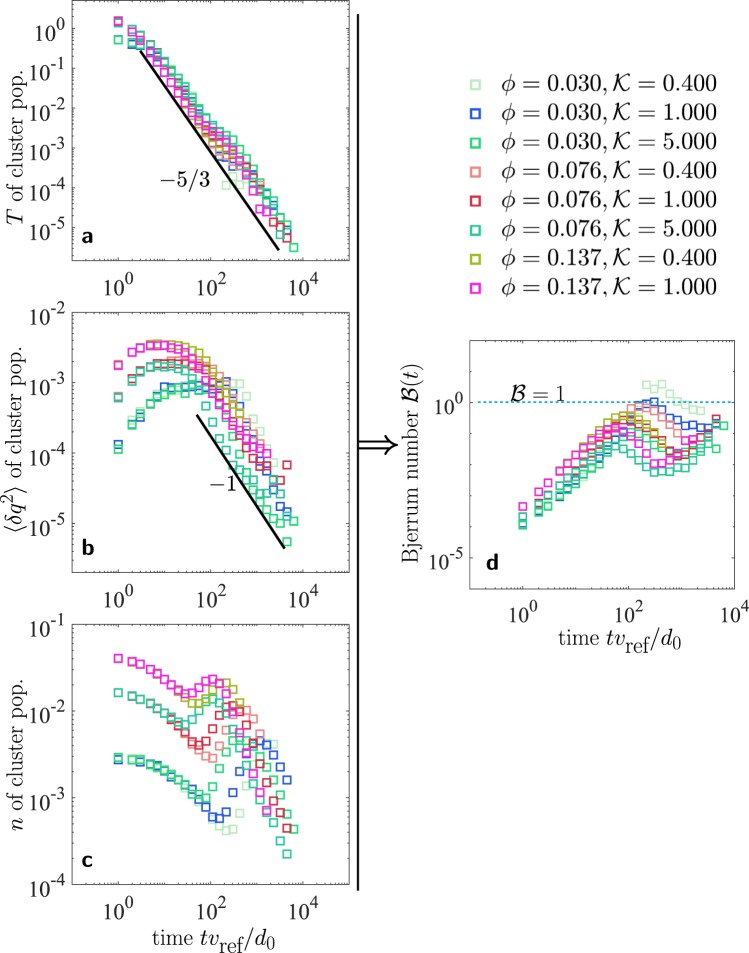
Evolution of (**a**) temperature $T$ of cluster population, (**b**) charge variance $\langle \delta {q}^{2}\rangle$ of cluster population, and (**c**) number density $n$ of cluster population, for different monomer filling fractions $\varphi$ and charge strength ${\mathscr{K}}$. (**d**) The granular temperature, charge variance and average size of the cluster population during aggregation evolve in such a manner that their non-dimensional combination ${\mathcal B} (t)={k}_{e}\langle \delta {q}^{2}\rangle /(Td)\le 1$ (see also Fig. 4). Both temperature and charge variance of cluster population decay as power laws (with exponents marked as legends). The number density evolution, however, is highly dynamic and exhibits a non-monotonic behavior due to emergence of mesoscopic flow (see Tables 2 and 3 for $\varphi$, ${\mathscr{K}}$, ${v}_{{\rm{ref}}}$, ${d}_{0}$).

The time evolution of *T*, $\langle \delta {q}^{2}\rangle$ and *n* of aggregate population from MD simulations is shown in Fig. 3. The aggregate temperature from simulations is  computed as $\frac{3}{2}T=\frac{1}{{N}_{{\rm{agg}}}}\,{\sum }_{k}\,\frac{1}{2}{m}_{k}[({{\bf{v}}}_{k}-{\bf{V}}{)}^{2}]$. Notice that **v**_*k*_ and *m*_*k*_ are center of mass velocity, and mass of the *k*^*th*^ aggregate respectively, and should not be confused with monomer velocities and masses. **V** is the local advective velocity in the neighborhood of *k*^*th*^ aggregate. Similarly, $\langle \delta {q}^{2}\rangle =\frac{1}{{N}_{{\rm{a}}{\rm{g}}{\rm{g}}}}\,{\sum }_{k}\,{({q}_{k}-{q}_{{\rm{m}}{\rm{e}}{\rm{a}}{\rm{n}}})}^{2}$, and $n={N}_{{\rm{agg}}}/V$, where *N*_agg_ is the total number of aggregates and *V* is the system volume. In the MD simulations, oppositely charged monomers hold together into mechanical contact when the elastic repulsion between them balances the Coulomb attraction. This is the scenario at low granular temperature when the dissipative force term, and the inertial term, in the equation of motion [Eq. (56)] is negligible compared to the elastic and Coulomb terms. We identify the aggregates with the following condition: if the distance between the centers of two monomers *r*_*ij*_ is less than or equal to the monomer diameter *d*_0_, they belong to the same aggregate. Once all the aggregates in the system are identified using the above definition, the aggregate velocity and mass as a whole is computed. If *v*_*i*_ are the velocities of monomers in *k*^*th*^ aggregate, the aggregate velocity *v*_*k*_ is computed by ${v}_{k}={\sum }_{i}\,{m}_{i}{v}_{i}/{\sum }_{i}\,{m}_{i}$, which is the center of mass velocity of the aggregate. The mass of the aggregate is simply ${m}_{k}={\sum }_{i}\,{m}_{i}$. Similarly, the net charge on *k*^*th*^ aggregate is ${q}_{k}={\sum }_{i}\,{q}_{i}$.

Initially ($t{v}_{{\rm{ref}}}/{l}_{{\rm{ref}}} < {10}^{2}$), the relative monomer collision velocities *v*_*ij*_ remain larger than the time varying threshold $\sqrt{b}\equiv \sqrt{\frac{{k}_{e}|{q}_{i}{q}_{j}|}{2Td}}$ (see Methods for the treatment of threshold *b* in the kinetic theory). In this time regime, the collisions are primarily restitutive, leading to either Coulomb scattering without collision, or charge exchange and dissipation without considerable aggregation. The dissipation reduces *T* [Fig. 3(a)] while the charge exchange increases $\langle \delta {q}^{2}\rangle$ [Fig. 3(b)]. In this time regime the number density *n*, and thus size *d* of the aggregates, is altered only moderately due to those low velocity attractive monomer encounters which lead to aggregation [Fig. 3(c)].

As a result of our kinetic formulation, the dynamics of *n*, $T$ and $\langle \delta {q}^{2}\rangle$ can be collected into the evolution of ${\mathcal B}$, shown in Fig. 3(d). The Bjerrum number ${\mathcal B}$ initially increases, which indicates that temperature decreases at a faster rate than the rate of increase of charge variance and the aggregate size. As the relative velocities *v*_*ij*_ approach the threshold $\sqrt{b}$, ${\mathcal B} \to 1$. Near this time, the dynamics cross over to aggregative collapse. The individual particles, or monomers, cluster in such a way that the charge variance of the cluster population now begins to reduce. The temperature of the aggregate population keeps decreasing at the same rate with a slight dip near the aggregative collapse. The number density starts to evolve non-monotonically. We explore the non-monotonicity in the next section. These results are robust under variation of the initial monomer filling fraction $\varphi$ and the charge strength ${\mathscr{K}}$ [Fig. 3].

**Figure 4:**
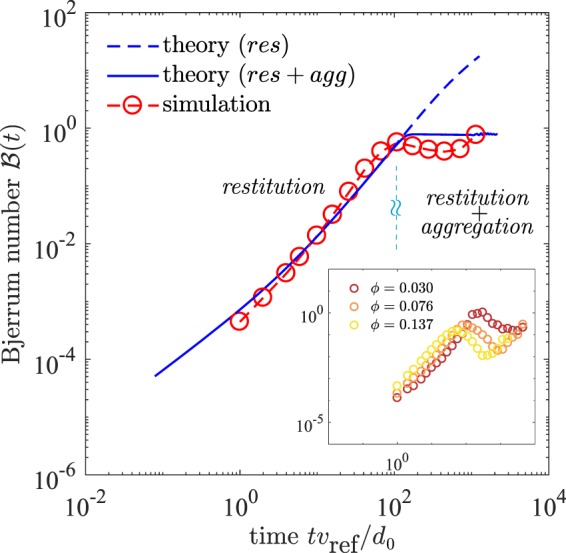
The granular temperature, charge variance and average size of the cluster population during aggregation evolve in such a manner that their non-dimensional combination ${\mathcal B} (t)={k}_{e}\langle \delta {q}^{2}\rangle /(Td)\le 1$. This is not captured in the kinetic theory if only restitutive (no aggregation) collisions are considered (dashed line). The granular MD simulations (symbols) confirm the analytical results. (inset for different monomer filling fraction $\varphi$).

After the initial time regime and the aggregative collapse, the crossover in the dynamics is depicted in Fig. 4, where the evolution of ${\mathcal B}$, and its comparison with the solution of Eqs (6–8) is highlighted. We solve the full kinetic equations Eqs (6–8) including the aggregation kinetics [Fig. 4 (solid line)]. We also solve Eqs (6–8) for a system with only dissipative collisions and without aggregation; hence, the cluster size *d* remains unchanged. These results are shown in Fig. 4 (dashed line). In this limit of only restitutive kinetics, ${\mathcal B}$ increases continuously above the limiting value 1. The purely restitutive kinetic theory thus fails to predict the MD results. When aggregation is explicitly treated (solid line), the theory predicts an upper limit during the growth. The theory shows that once aggregation sets in, the aggregating granular gas obeys the constraint

11 $$ {\mathcal B} (t)\le 1.$$

The upper physical limit predicted in the theory, ${\mathcal B} (t) < 1$, is endorsed by the granular MD simulations under moderate variation of $\varphi$ and ${\mathscr{K}}$. It is notable that at later times, the limit ${\mathcal B} \le 1$ allows the right hand sides of the equations for number density, temperature and charge variance [Eqs (6–8)] to remain real valued during the aggregation process. This mathematical indication confirms the effectiveness of the quasi-monodisperse picture [Fig. 2] considered in the present study.

The initiation of aggregation brings about a power law decay in the charge variance [Fig. 3(b)]. It is notable that a different charge exchange model might provide a different charge buildup rate during the purely dissipative (restitutive) phase. However, the decay of charge variance during the aggregative phase is not expected to be influenced by charge exchange mechanism. The reason is that aggregation sets in at relatively low temperature where the motion of monomers, if any, inside the clusters is significantly decreased, and thus collisional charge-exchange is expected to be negligible. If two oppositely charged particles *i* and *j* collide with a speed which is below the aggregation threshold speed, it is considered that they form a single aggregate with net charge ${q}_{i}+{q}_{j}$ on it [Fig. 2]. Thus, the charge variance of the cluster population is reduced by the aggregation process, rather than by the collisional charge exchange.

Our key theoretical finding is that after the aggregative collapse, the decay of the charge variance of aggregate population and the growth of the size of the aggregates is balanced by the decay of temperature during the aggregation, resulting in the stationary value of ${\mathcal B} (t)$. The constraint ${\mathcal B} \le 1$ is robust in the theory, while the granular MD simulations suggest ${\mathscr{B}}(t)\lesssim 1$ and confirm the upper limit of ${\mathcal B} (t)$. It is also intriguing that the temperature of the cluster population still closely follows modified Haff’s law for different $\varphi$ and ${\mathscr{K}}$, despite complex heterogeneous aggregation-fragmentation events and the long-range electrostatic interactions.

The number density’s temporal evolution obtained from the MD simulations reveals a more intricate non-monotonic dynamics.  Initially  it decreases during small aggregate formations due to low velocity attractive monomer encounters. In an intermediate time regime, the emergence of mesoscopic particle fluxes triggers fragmentation events and the aggregate numbers increase. We quantify the emergence of mesoscopic flow using the Mach number (see Methods and Media therein). After this intermediate time regime, the aggregation again takes over and the number density of clusters starts to decrease. The non-monotonic evolution of *n* causes a dip in ${\mathcal B} (t)$ after the aggregative collapse ($t{v}_{{\rm{ref}}}/{l}_{{\rm{ref}}} > {10}^{2}$) [Figs 3 and 4]. For temperature values in this regime, the charge transfer events between monomers are statistically ineffective, and the evolution of $\langle \delta {q}^{2}\rangle$ is primarily dominated by aggregation events. We find that a maximum of charge fluctuations $\langle \delta {q}^{2}\rangle$ occurs near this crossover.

The size difference between aggregates in the MD simulations further adds to the complexity of ${\mathcal B}$’s evolution after the aggregative collapse, which is neglected in the homogeneous and quasi-monodisperse aggregation kinetic theory. However, the theory still clearly predicts the growth of ${\mathcal B}$ and selects a unique upper limit after the aggregative collapse. To further explore the mechanisms behind the non-monotonic evolution of *n*, we explore the spatially heterogeneous cluster dynamics and nature of the structures from the MD simulations.

### Inhomogeneous aggregation and fractal growth

To gain access to the spatial structure formation in the gas, we perform a detailed cluster analysis of the results from granular MD simulation, see Fig. 1.

**Figure 5:**
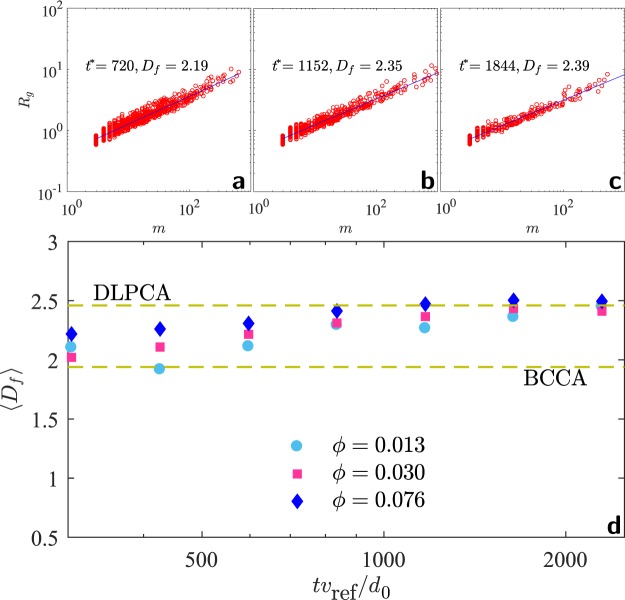
(**a**–**c**) The scaling between cluster mass $m$ and their radius of gyration ${R}_{g}$, $m\sim {R}_{g}^{{D}_{f}}$ at different times, and (**d**) the time evolution of $\langle {D}_{f}\rangle$, thus obtained, for different filling fractions. The average fractal dimension in the aggregating charged gas varies  between the average values reported for ballistic cluster-cluster aggregation (BCCA, $\langle {D}_{f}\rangle \simeq 1.94$) and diffusion-limited particle-cluster aggregation (DLPCA, $\langle {D}_{f}\rangle \simeq 2.46$)^6,33^. ${t}^{\ast }\equiv t{v}_{{\rm{ref}}}/{d}_{0}$.

The morphology of the aggregates is studied by computing the average fractal dimension $\langle {D}_{f}\rangle$^31,32^ of cluster population from the scaling relation $m\sim {R}_{g}^{\langle {D}_{f}\rangle }$ between cluster masses *m*, and radii of gyration ${R}_{g}={[\frac{1}{{N}_{{\rm{mon}}}}{\sum }_{i}{({{\bf{r}}}_{i}-{{\bf{r}}}_{{\rm{mean}}})}^{2}]}^{1/2}$, where the index *i* runs over total number of monomers *N*_mon_ in a given aggregate. Here **r**_*i*_ is the position vector  of the *i*^*th*^ monomer in the given aggregate and **r**_mean_ is the position vector  of the center of mass of that aggregate. Once *m* and *R*_*g*_ of all the aggregates in the system are calculated, we compute the *R*_*g*_ versus *m* scatter-plots at different times, for example in Fig. 5(a–c). We repeat this for different times, and calculate $\langle {D}_{f}\rangle$ as the slope of the fit to ${R}_{g}$ versus $m$ scatter-plots. Figure 5(d) shows the time evolution of the exponent $\langle {D}_{f}\rangle$ for  different filling fraction. The average fractal dimension lies between the average values reported for the ballistic cluster-cluster aggregation (BCCA, $\langle {D}_{f}\rangle \simeq 1.94$) and the diffusion-limited particle-cluster aggregation (DLPCA, $\langle {D}_{f}\rangle \simeq 2.46$)^6,33^ models. In time, $\langle {D}_{f}\rangle$ is dynamic and changes across the two model limits. These results indicate that the aggregate structures retain their fractal nature over time.

The BCCA and DLPCA are popular models for aggregation that have been used for neutral dust agglomeration (e.g. hit and stick, ballistic motion^6^), wet granulate aggregation (sticking due to capillary bridges and ballistic motion^16^), colloidal aggregation (van der Waals and repulsions^34^), and hit and stick agglomeration in Brownian particles under frictional drag^35^. The observation that $\langle {D}_{f}\rangle$ lies between the reported average values of $\langle {D}_{f}\rangle$ for BCCA and DLPCA indicates the presence of mixed characteristics from both of these simplified models. The size distribution in an aggregating, charged granular gas^25^ tends to resemble a DLPCA-like behavior where the smaller size aggregates are larger in number, in contrast to a BCCA-like model where the size distribution is typically bell-shaped^6^. On the other hand, the monomer motion is found to be highly sub-diffusive^25^ in agreement with the BCCA model. In addition, the Coulombic interactions will cause considerable deviations from the short-ranged or ballistic propagation typical of the BCCA or DLPCA models. We find that the long-range forces due to a bipolar charge distribution lead to the value of $\langle {D}_{f}\rangle$ intermediate between the above two aggregation models, indicated by dashed lines in Fig. 5(d).

### Interplay between fractals and mesoscopic flow

Apart from the long-range effects, the morphology of the aggregates is also altered by additional mechanisms. We discuss two physical processes that are not captured in the analytical theory, but that we investigate via our MD simulations.

First, in our modified Boltzmann kinetic description, the collisions between aggregates at any given time are considered as collisions between two spheres with sizes equal to the average size of the aggregate population. This is a quasi-monodisperse assumption typically used in cluster-cluster aggregation models. The quasi-monodispersity however neglects the morphology and surface irregularities of the colliding aggregates. In practice, collisions between two aggregates with large size difference are also possible. Additionally, individual monomers might collide with large aggregates and result in fragmentation events. Our kinetic description neglects these specific scenarios. It is important to note that the main reason for the mismatch between kinetic theory and MD results for the density evolution is our neglect of size differences among aggregates in the kinetic theory.

Secondly, granular gases are characterized by the emergence of an advective flow^27,36^ which we find, in the present case, induces the non-monotonicity in the temporal evolution of the number density [Fig. 3(c)]. Due to the advective flow, aggregates which are weakly connected are prone to fragmentation. This results in an intermediate regime where the concentration of aggregates increases instead of decreasing.

Excluding the two above mechanisms explains the slight deviation of our quasi-monodisperse Boltzmann theory from the non-monotonic behavior of ${\mathcal B} (t)$ after the crossover to aggregative collapse.

## Discussion

We have derived the rate equations for the evolution of the number density $n$, granular temperature $T$, and charge variance $\langle \delta {q}^{2}\rangle$ of the cluster population in a collisionally charged, aggregating granular gas. In contrast to well-known Smoluchowski-type equations, we have explicitly coupled $n$ to the decay of $T$ and charge variance. We have compared the results with three-dimensional molecular dynamics simulations and the outcomes of a detailed cluster analysis, and have explored the morphology of the aggregating structures via fractal dimension.

Taken together, our results indicate that the aggregation process in a charged granular gas is quite dynamic, while respecting some physical constraints. The growth process obeys ${\mathcal B} (t)={k}_{e}\langle \delta {q}^{2}\rangle /(Td)\le 1$, while morphologically, the clusters exhibit statistical self-similarity, persistent over time during the growth. The fractal dimension and growth of structures is intermediate between the BCCA and DLPCA models. We also demonstrate that the application of a purely dissipative kinetic treatment is not sufficient to make predictions about global observables such as $T$ and $\langle \delta {q}^{2}\rangle$ in an aggregating charged granular gas.

Finally, we believe that our kinetic approach can be applied to study aggregation processes in systems such as wet granulates with ion transfer mechanism^37,38^, dissipative cell or active particle collections under long-range hydrodynamic and electrostatic effects^39,40^, and charged ice-ice collisions^41^.

## Methods

### Kinetics and modified collision integral

After obtaining the number of direct collisions ${N}_{c}^{-}$ in Eq. (2) in the main text, let us consider the number of particles ${N}_{c}^{+}$ per unit spatial volume having initial velocity-charge values $({{\bf{v}}}_{i}^{{\rm{^{\prime} }}{\rm{^{\prime} }}},{q}_{i}^{{\rm{^{\prime} }}{\rm{^{\prime} }}})$ and $({{\bf{v}}}_{j}^{{\rm{^{\prime} }}{\rm{^{\prime} }}},{q}_{j}^{{\rm{^{\prime} }}{\rm{^{\prime} }}})$ in the intervals $d{{\bf{v}}}_{i}^{{\rm{^{\prime} }}{\rm{^{\prime} }}}d{q}_{i}^{{\rm{^{\prime} }}{\rm{^{\prime} }}}$ and $d{{\bf{v}}}_{j}^{{\rm{^{\prime} }}{\rm{^{\prime} }}}d{q}_{j}^{{\rm{^{\prime} }}{\rm{^{\prime} }}}$ which, post-collision, enter the intervals $d{{\bf{v}}}_{i}d{q}_{i}$ and $d{{\bf{v}}}_{j}d{q}_{j}$ in time Δ*t*

12 $${N}_{c}^{+}={f}_{i}^{{\rm{^{\prime} }}{\rm{^{\prime} }}}{d{\bf{v}}}_{i}^{{\rm{^{\prime} }}{\rm{^{\prime} }}}d{q}_{i}^{{\rm{^{\prime} }}{\rm{^{\prime} }}}{f}_{j}^{{\rm{^{\prime} }}{\rm{^{\prime} }}}{d{\bf{v}}}_{j}^{{\rm{^{\prime} }}{\rm{^{\prime} }}}d{q}_{j}^{{\rm{^{\prime} }}{\rm{^{\prime} }}}|{{\bf{v}}}_{ij}^{{\rm{^{\prime} }}{\rm{^{\prime} }}}\cdot {\bf{n}}|{\rm{\Theta }}(-{{\bf{v}}}_{ij}^{{\rm{^{\prime} }}{\rm{^{\prime} }}}\cdot {\bf{n}}){{\rm{\Theta }}}_{q}^{{\rm{^{\prime} }}{\rm{^{\prime} }}}d{\sigma }^{{\rm{^{\prime} }}{\rm{^{\prime} }}}{\rm{\Delta }}t,$$

and thus the net increase of number of particles per unit time and volume is ${N}_{c}^{+}-{N}_{c}^{-}$. We can relate the primed velocities to the unprimed via

13 $${d{\bf{v}}}_{i}^{{\rm{^{\prime} }}{\rm{^{\prime} }}}d{{\bf{v}}}_{j}^{{\rm{^{\prime} }}{\rm{^{\prime} }}}={J}_{ij}^{v}d{{\bf{v}}}_{i}d{{\bf{v}}}_{j},$$

where ${J}_{ij}^{v}=1+(6/5){C}_{\epsilon }{v}_{ij}^{1/5}+\ldots$ is the Jacobian of the transformation for viscoelastic particles^27^. Here ${C}_{\epsilon }$ is a material constant. To obtain the transformation $d{q}_{i}^{{\rm{^{\prime} }}{\rm{^{\prime} }}}d{q}_{j}^{{\rm{^{\prime} }}{\rm{^{\prime} }}}\to d{q}_{i}d{q}_{j}$, we consider the ratio of relative charges after and before the collision

14 $$r=\frac{{q}_{i}-{q}_{j}}{{q}_{i}^{{\rm{^{\prime} }}{\rm{^{\prime} }}}-{q}_{j}^{{\rm{^{\prime} }}{\rm{^{\prime} }}}},$$

and in addition we impose charge conservation during collisions

15 $${q}_{i}+{q}_{j}={q}_{i}^{{\rm{^{\prime} }}{\rm{^{\prime} }}}+{q}_{j}^{{\rm{^{\prime} }}{\rm{^{\prime} }}}.$$

The above two relations finally provide the transformation

16 $${dq}_{i}^{{\rm{^{\prime} }}{\rm{^{\prime} }}}d{q}_{j}^{{\rm{^{\prime} }}{\rm{^{\prime} }}}={J}_{ij}^{q}d{q}_{i}d{q}_{j},$$

where, for example, ${J}_{ij}^{q}=\frac{2}{r}$ for a constant *r*. This means that the differential charge-space volume element shrinks or expands by a factor of *r*/2. In general, the charge transfer may depend on pre-collision velocities and charges, and the expressions of *r* and ${J}_{ij}^{q}$ can be quite complicated. The charge exchange depends on myriad factors such as size, composition, and crystalline properties. Incorporating the above phase-space volume transformations due to collisions, the net change Δ*N*_*c*_ of number of particles per unit phase-space volume and in time Δ*t* reads

$${\rm{\Delta }}{N}_{c}=(\frac{1}{\epsilon ({v}_{ij})}{J}_{ij}^{v}{J}_{ij}^{q}{f}_{i}^{{\rm{^{\prime} }}{\rm{^{\prime} }}}{f}_{j}^{{\rm{^{\prime} }}{\rm{^{\prime} }}}-{f}_{i}{f}_{j})|{{\bf{v}}}_{ij}\cdot {\bf{n}}|{\rm{\Theta }}(-{{\bf{v}}}_{ij}\cdot {\bf{n}})d{{\bf{v}}}_{j}d{q}_{j}d\sigma {{\rm{\Theta }}}_{q}{\rm{\Delta }}t,$$

where we assume that the differential cross-section and the contact condition specified by ${{\rm{\Theta }}}_{q}$ are the same for direct and inverse collisions. Finally, dividing by Δ*t*, and integrating over all incoming particle velocities and charges from all directions in the limit ${\rm{\Delta }}t\to 0$, we obtain a formal expression for the collision integral

17 $${I}_{{\rm{c}}{\rm{o}}{\rm{l}}{\rm{l}}}=\int (\frac{1}{\epsilon ({v}_{ij})}{J}_{ij}^{v}{J}_{ij}^{q}{f}_{i}^{{\rm{^{\prime} }}{\rm{^{\prime} }}}{f}_{j}^{{\rm{^{\prime} }}{\rm{^{\prime} }}}-{f}_{i}{f}_{j})|{{\bf{v}}}_{ij}\cdot {\bf{n}}|{\rm{\Theta }}(-{{\bf{v}}}_{ij}\cdot {\bf{n}})d{{\bf{v}}}_{j}d{q}_{j}d\sigma {{\rm{\Theta }}}_{q}.$$

At this point the particle encounters which do not lead to a physical contact have been excluded using ${{\rm{\Theta }}}_{q}$, however, collisions that lead to aggregation have not been explicitly accounted. We do so by taking $\epsilon =0$ for certain conditions on the relative velocity ${v}_{ij}$, and by considering the charge transferred to particle $i$ equal to the charge on particle $j$ [Eqs (25–30) below]. In *I*_coll_, distant encounters, which do not lead to a contact between particles (glancing collisions) are neglected and the charge exchange and dissipation is considered only during the contact. The long-range effect is incorporated via collision cross-section [Eq. (38)].

### Splitting restitution and aggregation

The time rate of change of the average of a microscopic quantity $\psi ({{\bf{v}}}_{i},{q}_{i})$ is obtained by multiplying the Boltzmann equation for ${f}_{i}$ by ${\psi }_{i}$ and integrating over ${{\bf{v}}}_{i},{q}_{i}$, *i*.*e*.

18 $$\frac{\partial \langle \psi \rangle }{\partial t}=\int \,d{{\bf{v}}}_{i}d{q}_{i}{\psi }_{i}\frac{\partial {f}_{i}}{\partial t}=\int \,d{{\bf{v}}}_{i}d{q}_{i}{\psi }_{i}{I}_{{\rm{coll}}}\mathrm{.}$$

It can be shown that

19 $$\begin{array}{rcl}\frac{\partial \langle \psi \rangle }{\partial t} & = & \int \,d{{\bf{v}}}_{i}d{q}_{i}{\psi }_{i}{I}_{{\rm{coll}}}\\  & = & \frac{1}{2}\,\int \,d{{\bf{v}}}_{i}d{{\bf{v}}}_{j}d{q}_{i}d{q}_{j}d\sigma {f}_{i}{f}_{j}|{{\bf{v}}}_{ij}\cdot {\bf{n}}|{\rm{\Theta }}(\,-\,{{\bf{v}}}_{ij}\cdot {\bf{n}}){{\rm{\Theta }}}_{q}{\rm{\Delta }}[{\psi }_{i}+{\psi }_{j}]\\  & = & \int \,d{{\bf{v}}}_{i}d{{\bf{v}}}_{j}d{q}_{i}d{q}_{j}d\sigma {f}_{i}{f}_{j}|{{\bf{v}}}_{ij}\cdot {\bf{n}}|{\rm{\Theta }}(\,-\,{{\bf{v}}}_{ij}\cdot {\bf{n}}){{\rm{\Theta }}}_{q}{\rm{\Delta }}[{\psi }_{i}],\end{array}$$

where ${\rm{\Delta }}[{\psi }_{i}+{\psi }_{j}]=({\psi }_{i}^{{\rm{^{\prime} }}}+{\psi }_{j}^{{\rm{^{\prime} }}}-{\psi }_{i}-{\psi }_{j})$ and ${\rm{\Delta }}[{\psi }_{i}]=({\psi }_{i}^{{\rm{^{\prime} }}}-{\psi }_{i})$ is the change of $\psi$ during the collision between pair *i*, *j*, and the prime denotes a post collision value *I*_coll_. We consider the number density, the kinetic energy or granular temperature, and the charge variance (the system is globally neutral and the mean charge variation $\langle \delta q\rangle$ is zero), respectively

20 $$(i)\,\psi =n,$$

21 $$(ii)\,\psi =\frac{1}{2}m{v}^{2},$$

22 $$(iii)\,\psi ={(\delta q)}^{2}={(q-{q}_{0})}^{2}={(q-\frac{{q}_{i}+{q}_{j}}{2})}^{2},$$

where ${q}_{0}=\frac{{q}_{i}+{q}_{j}}{2}$ is the mean charge on the colliding pair. At this point we differentiate the restitutive or dissipative collisions from aggregative ones by splitting ${{\rm{\Theta }}}_{q}{\rm{\Delta }}[{\psi }_{i}]$ as

23 $$\begin{array}{rcl}{{\rm{\Theta }}}_{q}{\rm{\Delta }}[{\psi }_{i}] & = & {{\rm{\Delta }}}^{res}[{\psi }_{i}]{\rm{\Theta }}({v}_{ij}-\sqrt{b}),\\  &  & +\,{{\rm{\Delta }}}^{agg}[{\psi }_{i}]{\rm{\Theta }}(\,-\,{q}_{i}{q}_{j}){\rm{\Theta }}(\sqrt{b}-{v}_{ij}),\end{array}$$

where

24 $$\sqrt{b}\equiv \sqrt{\frac{2{k}_{e}|{q}_{i}{q}_{j}|}{md}}.$$

If ${v}_{ij} > \sqrt{b}$, the particles collide and separate after the collision irrespective of the sign of ${q}_{i}{q}_{j}$ (attractive or repulsive). This leads to dissipation of energy with finite non-zero $\epsilon =\epsilon ({v}_{ij})$, and charge exchange according to a specified rule. The aggregative part is zero in this case. If ${v}_{ij} < \sqrt{b}$ and ${q}_{i}{q}_{j} < 0$ (attractive encounters at low velocities), the particles collide and aggregate with $\epsilon =0$, and with charge exchange to particle $i$ equal to ${q}_{j}$. If ${v}_{ij} < \sqrt{b}$ and ${q}_{i}{q}_{j} > 0$ (repulsive encounters at low velocities), no physical contact takes place between the particles which leads to neither dissipation nor aggregation (${{\rm{\Theta }}}_{q}{\rm{\Delta }}[{\psi }_{i}]=0$). Also represented schematically in Fig. 2 in the main text.

The expressions for ${{\rm{\Delta }}}^{res}[{\psi }_{i}]$ and ${{\rm{\Delta }}}^{agg}[{\psi }_{i}]$ are obtained as follows. The particle number does not change during a dissipative collision but reduces by one in an aggregative collision, *i*.*e*.

25 $$\begin{array}{ccc}{{\rm{\Delta }}}_{n}^{res}[{\psi }_{i}+{\psi }_{j}] & = & 0,\\ {{\rm{\Delta }}}_{n}^{agg}[{\psi }_{i}+{\psi }_{j}] & = & -\,1.\end{array}$$

For the granular temperature,

26 $$\begin{array}{ccc}{{\rm{\Delta }}}_{T}^{res}[{\psi }_{i}+{\psi }_{j}] & = & -\,\frac{1}{2}m(1-{\epsilon }^{2})\,{({{\bf{v}}}_{ij}\cdot {\bf{n}})}^{2},\\ {{\rm{\Delta }}}_{T}^{agg}[{\psi }_{i}+{\psi }_{j}] & = & -\,\frac{1}{2}m{({{\bf{v}}}_{ij}\cdot {\bf{n}})}^{2},\end{array}$$

where we take the limit $\epsilon =0$ for the aggregation. The change in the charge variance is obtained as

27 $$\begin{array}{ccc}{{\rm{\Delta }}}_{q}^{res}[{\psi }_{i}] & = & (\delta {q}_{i}^{2}{)}^{{\rm{^{\prime} }}}-(\delta {q}_{i}^{2})\\  & = & {({q}_{i}^{{\rm{^{\prime} }}}-{q}_{i})}^{2}+2({q}_{i}^{{\rm{^{\prime} }}}-{q}_{i})\,({q}_{i}-{q}_{0}),\end{array}$$

where $({q}_{i}^{{\rm{^{\prime} }}}-{q}_{i})$ equals the charge transferred to particle *i* during its collision with particle $j$. For the charge transfer, based on seminal experiments^10^, we consider

28 $$({q}_{i}^{{\rm{^{\prime} }}}-{q}_{i})={C}_{{\rm{\Delta }}q}|{{\bf{v}}}_{ij}\cdot {\bf{n}}{|}^{\eta }\frac{{q}_{i}-{q}_{0}}{|{q}_{i}-{q}_{0}|},$$

which is also obtainable if the charge transferred is considered proportional to the maximum contact area during the course of a collision^43^. In the present simulations, the value of the exponent $\eta =2\times 0.8$ after the experiments^10,42^, where a power law dependence was found of the charge exchange on the relative collision energy, when silica particles were impacted on a surface. In the MD simulations, we have verified that while moderate changes of $\eta$ around the experimental value might produce changes in the charge buildup rate of the gas (${t}^{\ast }\lesssim {10}^{2}$ in Fig. 3(b)), the dynamics of charge in the aggregation phase are unaffected (${t}^{\ast } > {10}^{2}$ in Fig. 3(b)). Once the magnitude of collision velocities $|{{\bf{v}}}_{ij}\cdot {\bf{n}}|$ reduces with reducing granular temperature, the value of $\eta$ has negligible influence on the decay rate of $\langle \delta {q}^{2}\rangle$, because it is primarily controlled by aggregation and merging of charges. The power law in Fig. 3(b) during the aggregation time regime remains unaffected.

Using Eq. (28) in Eq. (27), we  obtain

29 $${{\rm{\Delta }}}_{q}^{res}[{\psi }_{i}]={C}_{{\rm{\Delta }}q}^{2}|{{\bf{v}}}_{ij}\cdot {\bf{n}}{|}^{2\eta }+2{C}_{{\rm{\Delta }}q}|{{\bf{v}}}_{ij}\cdot {\bf{n}}{|}^{\eta }({q}_{i}-{q}_{0}\mathrm{).}$$

For aggregation, the charge tranferred to particle $i$ equals the charge on the merging particle $j$, *i*.*e*., ${q}_{i}^{{\rm{^{\prime} }}}-{q}_{i}={q}_{j}$, which gives

30 $$\begin{array}{rcl}{{\rm{\Delta }}}_{q}^{agg}[{\psi }_{i}] & = & (\delta {q}_{i}^{2})^{\prime} -(\delta {q}_{i}^{2})\\  & = & {q}_{i}{q}_{j}\mathrm{.}\end{array}$$

Putting Eqs (25, 26, 29 and 30) in (23), and then (23) in (19), the resulting integrals are solved, assuming the statistical independence of charge-velocity distribution function, *i*.*e*., $f({\bf{v}},q)=f({\bf{v}})f(q)$, and assuming that their scaled form remains Gaussian. In addition to the charge exchange, the coefficient of restitution is taken as velocity dependent^30,44^, *i*.*e*.

31 $$\epsilon =\epsilon (|{{\bf{v}}}_{ij}\cdot {\bf{n}}|)=1-{C}_{\epsilon }|{{\bf{v}}}_{ij}\cdot {\bf{n}}{|}^{1/5}+\ldots $$

while the long-range effects due to Coulomb interactions are taken into account by the change in collision cross section [Eq. (38)]. After integration we obtain Eqs (6–8) in the main text. The functions *g*_*k*_ in Eqs (6–8) have the forms

32 $${g}_{1}( {\mathcal B} )=[\frac{{C}_{agg}^{n}}{{l}_{1}^{3}{l}^{3}}]\,[{a}_{1}^{n}\,{\tan }^{-1}\,\frac{{l}_{1}}{ {\mathcal B} }+{a}_{2}^{n}+{a}_{3}^{n}\,{\tan }^{-1}\,\frac{ {\mathcal B} }{l}+{a}_{4}^{n}\,{\tan }^{-1}\,\frac{{l}_{1}}{ {\mathcal B} }+{a}_{5}^{n}+{a}_{6}^{n}\,{\tan }^{-1}\,\frac{l}{ {\mathcal B} }],$$

33 $${g}_{2}( {\mathcal B} )=[\frac{{C}_{res}^{T}}{{l}^{5}}]\,[{a}_{1}^{T}+{a}_{2}^{T}\,{\tan }^{-1}\,\frac{ {\mathcal B} }{l}],$$

34 $${g}_{3}( {\mathcal B} )=[\frac{{C}_{agg}^{T}}{{l}^{5}}]\,[{a}_{3}^{T}+{a}_{4}^{T}\,{\cot }^{-1}\,\frac{ {\mathcal B} }{l}+{a}_{5}^{T}+{a}_{6}^{T}\,{\tan }^{-1}\,\frac{ {\mathcal B} }{l}],$$

35 $${g}_{4}( {\mathcal B} )=[\frac{{C}_{res}^{q}}{{l}^{5}}]\,[{a}_{1}^{q}+{a}_{2}^{q}\,{\tan }^{-1}\,\frac{ {\mathcal B} }{l}],$$

36 $$\begin{array}{rcl}{g}_{5}( {\mathcal B} ) & = & [{C}_{agg}^{q}]\,[\frac{1}{{a}_{3}^{q}}({a}_{4}^{q}+{a}_{5}^{q}\,{\tan }^{-1}\,\frac{ {\mathcal B} }{{l}_{1}}+{a}_{6}^{q}\,{\tan }^{-1}\,\frac{l}{ {\mathcal B} })\\  &  & +\,\frac{1}{{a}_{7}^{q}}({a}_{8}^{q}\,{\tan }^{-1}\,\frac{{l}_{1}}{ {\mathcal B} }+{a}_{9}^{q}+{a}_{10}^{q}\,{\tan }^{-1}\,\frac{l}{ {\mathcal B} })],\end{array}$$

where the coefficients $l$, ${l}_{1}$, ${a}_{k}^{T}$, ${a}_{k}^{q}$, ${a}_{k}^{n}$ are functions of ${\mathcal B} (t)$ [Table 1].

### Derivation of the hydrodynamic equations (6–8)

To solve the integrals in Eq. (19) for different Δ$[{\psi }_{i}]$ from Eqs (25–30), we assume that the normalized velocity as well as charge distribution of the aggregating particle population at any time remain Gaussian, and the two are uncorrelated, *i*.*e*.

37 $$f({\bf{v}},q)=f({\bf{v}})f(q)=n{(\frac{m}{2\pi T})}^{\frac{3}{2}}{e}^{-m{v}^{2}\mathrm{/(2}T)}{(\frac{1}{2\pi \langle \delta {q}^{2}\rangle })}^{\frac{1}{2}}{e}^{-{q}^{2}\mathrm{/(2}\langle \delta {q}^{2}\rangle )}\mathrm{.}$$

**Figure 6:**
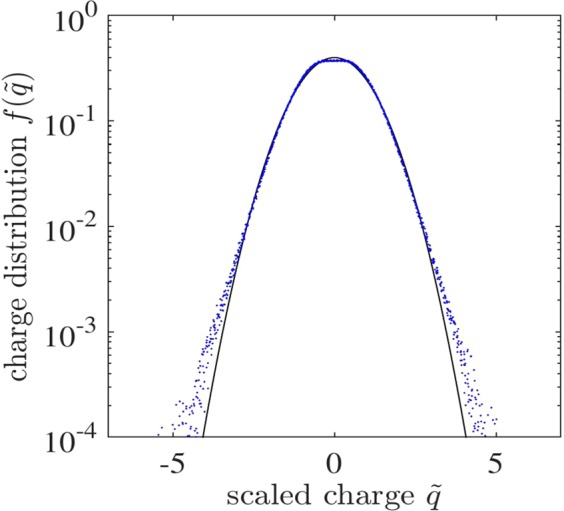
The scaled charge distribution $f(\tilde{q})$ of individual particles (monomers) obtained from typical MD simulation runs (dots) in the aggregated granular gas. The solid line is a Gaussian fit. Here $\tilde{q}=q/{\langle \delta {q}^{2}\rangle }^{\mathrm{1/2}}$.

In Fig. 6, we show the charge distribution of monomers obtained from typical simulation runs, which is essentially the distribution before initiation of the aggregation. In Eq. (37) above, we assume that although granular temperature *T* and charge variance $\langle \delta {q}^{2}\rangle$ do change with time due to restitution and aggregation, the shape of the scaled distribution remains close to Gaussian, and the increase of size/decrease of number of particles due to aggregation process does not alter the shape of the scaled distribution.

The attractive or repulsive long-range effects are emulated through an effective differential cross-section for a binary collision, which changes depending upon the sign and magnitude of charges on the particle pair *i*, *j* and their relative velocity, according to

38 $$d\sigma =(\frac{d\sigma }{d{\rm{\Omega }}})\,d{\rm{\Omega }}=\frac{{d}^{2}}{4}(1-\frac{2{k}_{e}{q}_{i}{q}_{j}}{dm|{{\bf{v}}}_{ij}\cdot {\bf{n}}{|}^{2}})\,d{\bf{n}}\approx \frac{{d}^{2}}{4}\,(1-\frac{2{k}_{e}{q}_{i}{q}_{j}}{dm{v}_{ij}^{2}})\,d{\bf{n}},$$

where $(\frac{d\sigma }{d{\rm{\Omega }}})$ is the differential cross-section per unit solid angle $d{\rm{\Omega }}\equiv d{\bf{n}}$. The expression $\frac{{d}^{2}}{4}(1-\frac{2{k}_{e}{q}_{i}{q}_{j}}{dm{v}_{ij}^{2}})$ is independent of **n**, and thus the total cross section is $\sigma$ = $\frac{{d}^{2}}{4}(1-\frac{2{k}_{e}{q}_{i}{q}_{j}}{dm{v}_{ij}^{2}})\,{\int }_{0}^{\pi }\,d\varphi d\theta \,\sin \,\theta$ = $\pi {d}^{2}(1-\frac{2{k}_{e}{q}_{i}{q}_{j}}{dm{v}_{ij}^{2}})$. For neutral particles, ${q}_{i}={q}_{j}=0$, and thus $\sigma =\pi {d}^{2}$. For ${q}_{i}{q}_{j} > 0$ (repulsive encounters), $\sigma  < \pi {d}^{2}$, while for ${q}_{i}{q}_{j} < 0$ (attractive encounters), $\sigma  > \pi {d}^{2}$. Thus Eq. (38) is a linear adjustment to the neutral cross-section and is reasonable approximation in case of small angle scattering^45^. The possibility of negative cross-section for repulsive encounters is eliminated by the function  ${\rm{\Theta }}({v}_{ij}-\sqrt{\frac{2{k}_{e}|{q}_{i}{q}_{j}|}{md}})$.

Below we explain the solution procedure for the restitutive, as well as aggregative, part of the equation for $\frac{\partial T}{\partial t}$. Similar procedure can be followed for the equations for $\frac{\partial n}{\partial t}$ and $\frac{\partial \langle \delta {q}^{2}\rangle }{\partial t}$.

Plugging Eq. (26) into Eq. (23) and then the resulting equation into Eq. (19), we find

39 $$\begin{array}{ccc}\frac{3}{2}\frac{{\rm{\partial }}T}{{\rm{\partial }}t} & = & {(\frac{3}{2}\frac{{\rm{\partial }}T}{{\rm{\partial }}t})}_{res}+{(\frac{3}{2}\frac{{\rm{\partial }}T}{{\rm{\partial }}t})}_{agg}\\  & = & \frac{1}{2}\,\int \,d{{\bf{v}}}_{i}d{{\bf{v}}}_{j}d{q}_{i}d{q}_{j}d\sigma {f}_{i}{f}_{j}|{{\bf{v}}}_{ij}\cdot {\bf{n}}|{\rm{\Theta }}(-{{\bf{v}}}_{ij}\cdot {\bf{n}})\\  &  & \times \,[-\frac{1}{2}m(1-{\epsilon }^{2})\,{({{\bf{v}}}_{ij}\cdot {\bf{n}})}^{2}]{\rm{\Theta }}({v}_{ij}-\sqrt{b})\\  &  & +\,\frac{1}{2}\,\int \,d{{\bf{v}}}_{i}d{{\bf{v}}}_{j}d{q}_{i}d{q}_{j}d\sigma {f}_{i}{f}_{j}|{{\bf{v}}}_{ij}\cdot {\bf{n}}|{\rm{\Theta }}(-{{\bf{v}}}_{ij}\cdot {\bf{n}})\\  &  & \times [-\frac{1}{2}m{({{\bf{v}}}_{ij}\cdot {\bf{n}})}^{2}]{\rm{\Theta }}(-{q}_{i}{q}_{j}){\rm{\Theta }}(\sqrt{b}-{v}_{ij}),\end{array}$$

which, after using the above Eqs (31 and 38), and separating the integrals over **n**, **v** and *q*, reads as

40 $$\begin{array}{rcl}\frac{3}{2}\frac{\partial T}{\partial t} & = & \frac{1}{2}\,{\int }_{q}\,d{q}_{i}d{q}_{j}f({q}_{i})f({q}_{j})\times {I}_{{\bf{v}}}^{res}\\  &  & +\,\frac{1}{2}\,{\int }_{q}\,{\rm{\Theta }}(\,-\,{q}_{i}{q}_{j})d{q}_{i}d{q}_{j}f({q}_{i})f({q}_{j})\times {I}_{{\bf{v}}}^{agg},\end{array}$$

where

41 $${I}_{{\bf{v}}}^{res}={\int }_{{\bf{v}}}\,{\rm{\Theta }}({v}_{ij}-\sqrt{b})d{{\bf{v}}}_{i}d{{\bf{v}}}_{j}f({v}_{i})f({v}_{j})\times \mathop{\overbrace{\frac{{d}^{2}}{4}(1-\frac{2{k}_{e}{q}_{i}{q}_{j}}{md|{{\bf{v}}}_{ij}{|}^{2}})}}\limits^{\frac{d\sigma }{d{\rm{\Omega }}}}\times {I}_{{\bf{n}}}^{res},$$

42 $${I}_{{\bf{v}}}^{agg}={\int }_{{\bf{v}}}\,{\rm{\Theta }}(\sqrt{b}-{v}_{ij})d{{\bf{v}}}_{i}d{{\bf{v}}}_{j}f({v}_{i})f({v}_{j})\times \frac{{d}^{2}}{4}(1-\frac{2{k}_{e}{q}_{i}{q}_{j}}{md|{{\bf{v}}}_{ij}{|}^{2}})\times {I}_{{\bf{n}}}^{agg},$$

and

43 $${I}_{{\bf{n}}}^{res}={\int }_{{\bf{n}}}\,d{\bf{n}}|{{\bf{v}}}_{ij}\cdot {\bf{n}}|{\rm{\Theta }}(-{{\bf{v}}}_{ij}\cdot {\bf{n}})(-m{C}_{\epsilon }|{{\bf{v}}}_{ij}\cdot {\bf{n}}{|}^{11/5}+\ldots ),$$

44 $${I}_{{\bf{n}}}^{agg}={\int }_{{\bf{n}}}\,d{\bf{n}}|{{\bf{v}}}_{ij}\cdot {\bf{n}}|{\rm{\Theta }}(\,-\,{{\bf{v}}}_{ij}\cdot {\bf{n}})(\,-\,\frac{1}{2}m|{{\bf{v}}}_{ij}\cdot {\bf{n}}{|}^{2}),$$

where in the aggregative part, we have set $\epsilon =0$, and ${\rm{\Theta }}(\,-\,{q}_{i}{q}_{j})$ selects only the attractive encounters against low velocities selected by ${\rm{\Theta }}(\sqrt{b}-{v}_{ij})$ -- the charge-velocity combination which leads to aggregation.

### Solution for the restitutive part ${(\frac{{\bf{3}}}{{\bf{2}}}\frac{{\boldsymbol{\partial }}{\boldsymbol{T}}}{{\boldsymbol{\partial }}{\boldsymbol{t}}})}_{{\boldsymbol{res}}}$

The solution for the parts ${I}_{{\bf{n}}}^{res}$, ${I}_{{\bf{v}}}^{res}$ are as follows.

46 $$\begin{array}{ccc}{I}_{{\bf{n}}}^{res} & = & {\int }_{{\bf{n}}}\,d{\bf{n}}|{{\bf{v}}}_{ij}\cdot {\bf{n}}|{\rm{\Theta }}(-{{\bf{v}}}_{ij}\cdot {\bf{n}})(-m{C}_{\epsilon }|{{\bf{v}}}_{ij}\cdot {\bf{n}}{|}^{11/5}+\ldots )\\  & = & {\int }_{0}^{\pi }\,{\int }_{\pi /2}^{\pi }\,d\varphi d\theta \,\sin \,\theta (-m{C}_{\epsilon }|{{\bf{v}}}_{ij}{|}^{16/5}|\,\cos \,\theta {|}^{16/5}+\ldots )\\  & = & -\,2\pi \frac{5}{21}m{C}_{\epsilon }|{{\bf{v}}}_{ij}{|}^{16/5}+\ldots \end{array}$$

Using Eqs (37 and 46) from the above text, ${I}_{{\bf{v}}}^{res}$ reads as

47 $$\begin{array}{ccc}{I}_{{\bf{v}}}^{res} & = & {\int }_{{\bf{v}}}\,{\rm{\Theta }}({v}_{ij}-\sqrt{b})d{{\bf{v}}}_{i}d{{\bf{v}}}_{j}[n{(\frac{m}{2\pi T})}^{3/2}{e}^{-(m/2T){v}_{i}^{2}}]\\  &  & \times \,[n{(\frac{m}{2\pi T})}^{3/2}{e}^{-(m/2T){v}_{j}^{2}}]\\  &  & \times \,[-2\pi \frac{5}{21}m{C}_{\epsilon }|{{\bf{v}}}_{ij}{|}^{16/5}]\frac{{d}^{2}}{4}(1-\frac{2{k}_{e}{q}_{i}{q}_{j}}{md|{{\bf{v}}}_{ij}{|}^{2}}).\end{array}$$

To perform the integration over the relative velocity ${{\bf{v}}}_{ij}$, the following transformations are made: (i) ${{\bf{w}}}_{ij}=\frac{2T}{m}({{\bf{v}}}_{i}-{{\bf{v}}}_{j})$, and (ii) ${{\bf{w}}}_{c}=\frac{2T}{m}({{\bf{v}}}_{i}+{{\bf{v}}}_{j})$, which also results in $d{{\bf{v}}}_{i}d{{\bf{v}}}_{j}=-\,\frac{1}{8}{(\frac{m}{4T})}^{-3}d{{\bf{w}}}_{ij}d{{\bf{w}}}_{c}$. Incorporating these transformations, we obtain

48 $$\begin{array}{ccc}{I}_{{\bf{v}}}^{res} & = & [-\frac{{n}^{2}{d}^{2}}{4}\frac{1}{8}{(\frac{m}{4T})}^{-3}{(\frac{m}{2\pi T})}^{3}{(\frac{4T}{m})}^{16/10}]\\  &  & \times \,{\int }_{{{\bf{w}}}_{ij}}\,d{{\bf{w}}}_{ij}[{\int }_{{{\bf{w}}}_{c}}\,d{{\bf{w}}}_{c}{e}^{-{w}_{c}^{2}}]{e}^{-{w}_{ij}^{2}}[-2\pi \frac{5}{21}m{C}_{\epsilon }{w}_{ij}^{16/5}]\,(1-\frac{{k}_{e}{q}_{i}{q}_{j}}{2Td{w}_{ij}^{2}})\\  & = & -\,2{T}^{8/5}[\frac{{2}^{1/5}{n}^{2}{d}^{2}}{{\pi }^{3}{m}^{8/5}}]\,\mathop{\underbrace{[4\pi \,{\int }_{0}^{{\rm{\infty }}}\,d{w}_{c}{w}_{c}^{2}{e}^{-{w}_{c}^{2}}]}}\limits_{{I}_{{w}_{c}}}\\  &  & \times \,\mathop{\underbrace{4\pi \,{\int }_{\sqrt{\frac{{k}_{e}|{q}_{i}{q}_{j}|}{2Td}}}^{{\rm{\infty }}}\,d{w}_{ij}{w}_{ij}^{2}{e}^{-{w}_{ij}^{2}}[-2\pi \frac{5}{21}m{C}_{\epsilon }{w}_{ij}^{16/5}](1-\frac{{k}_{e}{q}_{i}{q}_{j}}{2Td{w}_{ij}^{2}})}}\limits_{{I}_{{w}_{ij}}}.\end{array}$$

Notice the lower limit on relative velocities,

49 $$\sqrt{\frac{{k}_{e}|{q}_{i}{q}_{j}|}{2Td}}\equiv \sqrt{c},$$

is also altered due to the transformation ${{\bf{v}}}_{ij}\to {{\bf{w}}}_{ij}$. The integral ${I}_{{w}_{c}}$ gives

50 $${I}_{{w}_{c}}={\pi }^{3/2},$$

while the integral ${I}_{{w}_{ij}}$ is solved as

51 $$\begin{array}{ccc}{I}_{{w}_{ij}} & = & -\,\frac{40{\pi }^{2}}{21}m{C}_{\epsilon }\,{\int }_{\sqrt{\frac{{k}_{e}|{q}_{i}{q}_{j}|}{2Td}}}^{{\rm{\infty }}}\,d{w}_{ij}{w}_{ij}^{26/5}{e}^{-{w}_{ij}^{2}}(1-\frac{{k}_{e}{q}_{i}{q}_{j}}{2Td{w}_{ij}^{2}})\\  & = & -\,\frac{40{\pi }^{2}}{21}m{C}_{\epsilon }\,{\int }_{\sqrt{c}}^{{\rm{\infty }}}\,d{w}_{ij}{w}_{ij}^{26/5}{e}^{-{w}_{ij}^{2}}(1-\frac{a}{{w}_{ij}^{2}})\\  & = & -\,\frac{40{\pi }^{2}}{21}m{C}_{\epsilon }\frac{1}{2}[{\rm{\Gamma }}(\frac{31}{10},c)-a{\rm{\Gamma }}(\frac{21}{10},c)]\\  & \approx  & -\,\frac{40{\pi }^{2}}{21}m{C}_{\epsilon }\frac{1}{2}[{\rm{\Gamma }}(3,c)-a{\rm{\Gamma }}(2,c)]\\  & = & -\,\frac{40{\pi }^{2}}{21}m{C}_{\epsilon }\frac{1}{2}[2{e}^{-c}(1+c+{c}^{2})-a{e}^{-c}(1+c+{c}^{2})],\end{array}$$

where $a=\frac{{k}_{e}{q}_{i}{q}_{j}}{2Td}$ . Putting ${I}_{{w}_{c}}$, ${I}_{{w}_{ij}}$ in ${I}_{{\bf{v}}}^{res}$, and finally ${I}_{{\bf{v}}}^{res}$ in the restitutive part of Eq. (40), and integrating over ${q}_{i}$, ${q}_{j}$, we obtain

52 $$\begin{array}{ccc}{(\frac{3}{2}\frac{{\rm{\partial }}T}{{\rm{\partial }}t})}_{res} & = & \frac{1}{2}\,{\int }_{-{\rm{\infty }}}^{{\rm{\infty }}}\,{\int }_{-{\rm{\infty }}}^{{\rm{\infty }}}\,d{q}_{i}d{q}_{j}\\  &  & \times \,\frac{1}{\sqrt{2\pi \langle \delta {q}^{2}\rangle }}{e}^{-{q}_{i}^{2}/(2\langle \delta {q}^{2}\rangle )}\frac{1}{\sqrt{2\pi \langle \delta {q}^{2}\rangle }}{e}^{-{q}_{j}^{2}/(2\langle \delta {q}^{2}\rangle )}\\  &  & \times \,(-2){T}^{8/5}[\frac{{2}^{1/5}{n}^{2}{d}^{2}}{{\pi }^{3}{m}^{8/5}}]{\pi }^{3/2}\frac{(-40{\pi }^{2})}{21}m{C}_{\epsilon }\\  &  & \times \,\frac{1}{2}[2{e}^{-c}(1+c+{c}^{2})-a{e}^{-c}(1+c+{c}^{2})]\\  & = & -\,{T}^{8/5}[\frac{{C}_{res}^{T}}{{l}^{5}}]\,[{a}_{1}^{T}+{a}_{2}^{T}\,{\tan }^{-1}\,\frac{{\mathscr{B}}}{l}].\end{array}$$

Notice that if ${\mathcal B} =0$, we recover the classical Haff’s law for viscoelastic granular gas.

### Solution for the aggregative part ${(\frac{{\bf{3}}}{{\bf{2}}}\frac{{\boldsymbol{\partial }}{\boldsymbol{T}}}{{\boldsymbol{\partial }}{\boldsymbol{t}}})}_{{\boldsymbol{agg}}}$

The solution for the parts ${I}_{{\bf{n}}}^{agg}$, ${I}_{{\bf{v}}}^{agg}$ are as follows.

53 $$\begin{array}{rcl}{I}_{{\bf{n}}}^{agg} & = & {\int }_{{\bf{n}}}\,d{\bf{n}}|{{\bf{v}}}_{ij}\cdot {\bf{n}}|{\rm{\Theta }}(\,-\,{{\bf{v}}}_{ij}\cdot {\bf{n}})(\,-\,\frac{1}{2}m|{{\bf{v}}}_{ij}\cdot {\bf{n}}{|}^{2})\\  & = & {\int }_{0}^{\pi }\,{\int }_{\pi \mathrm{/2}}^{\pi }\,d\varphi d\theta \,\sin \,\theta |{{\bf{v}}}_{ij}||\,\cos \,\theta |(\,-\,\frac{1}{2}m|{{\bf{v}}}_{ij}{|}^{2}|\,\cos \,\theta {|}^{2})\\  & = & \frac{\pi }{4}m|{{\bf{v}}}_{ij}{|}^{3}\mathrm{.}\end{array}$$

Using Eqs (37 and 53) from the above text, and again using the variable transformations (i) ${{\bf{w}}}_{ij}=\frac{2T}{m}({{\bf{v}}}_{i}-{{\bf{v}}}_{j})$, (ii) ${{\bf{w}}}_{c}=\frac{2T}{m}({{\bf{v}}}_{i}+{{\bf{v}}}_{j})$, (iii) $d{{\bf{v}}}_{i}d{{\bf{v}}}_{j}=-\,\frac{1}{8}{(\frac{m}{4T})}^{-3}d{{\bf{w}}}_{ij}d{{\bf{w}}}_{c}$, the integral ${I}_{{\bf{v}}}^{agg}$ in (42) reduces to

54 $${I}_{{\bf{v}}}^{agg}=-\,{T}^{3}[\frac{2\sqrt{2}{\pi }^{2}{d}^{2}}{{m}^{2}}]\mathop{\underbrace{{\int }_{0}^{\sqrt{\frac{{k}_{e}|{q}_{i}{q}_{j}|}{2Td}}}\,d{w}_{ij}{w}_{ij}^{2}{w}_{ij}^{3}{e}^{-{w}_{ij}^{2}}(1-\frac{{k}_{e}{q}_{i}{q}_{j}}{2Td{w}_{ij}^{2}})}}\limits_{{I}_{{w}_{ij}}}\mathrm{.}$$

Here notice that now the relative velocity limits are from ${w}_{ij}=0$ to $\sqrt{c}$, the condition for aggregation selected by ${\rm{\Theta }}(\sqrt{c}-{w}_{ij})$. Finally putting (54) into aggregative part of (40) and integrating over *q*_*i*_, *q*_*j*_, we obtain

55 $${(\frac{3}{2}\frac{\partial T}{\partial t})}_{agg}={n}^{2}{T}^{\mathrm{3/2}}[\frac{{C}_{agg}^{T}}{{l}^{5}}]\,[{a}_{3}^{T}+{a}_{4}^{T}\,{\cot }^{-1}\,\frac{ {\mathcal B} }{l}+{a}_{5}^{T}+{a}_{6}^{T}\,{\tan }^{-1}\,\frac{ {\mathcal B} }{l}]\mathrm{.}$$

Notice that after integrating from ${w}_{ij}=0$ to $\sqrt{c}$, the integration over *q*_*i*_, *q*_*j*_ is to be broken into the sum of two parts, one over ${q}_{i}\in (\,-\,\infty ,0]$, ${q}_{j}\in [0,+\,\infty )$, plus a second integral over ${q}_{i}\in [0,+\,\infty )$, ${q}_{j}\in (\,-\,\infty ,0]$, to satisfy the aggregative condition set by ${\rm{\Theta }}(-{q}_{i}{q}_{j}){\rm{\Theta }}(\sqrt{c}-{w}_{ij})$.

A similar procedure is followed for ${(\frac{\partial \langle \delta {q}^{2}\rangle }{\partial t})}_{res}$, ${(\frac{\partial \langle \delta {q}^{2}\rangle }{\partial t})}_{agg}$ and ${(\frac{\partial n}{\partial t})}_{agg}$ using corresponding Δ$[{\psi }_{i}]$ (See Supplementary Mathematica script). Finally, the key constraint to be noted is that the solutions of the integrals in the rate equation for *T* are real valued for ${\mathcal B} \le 4$, while in equations for $\langle \delta {q}^{2}\rangle$ and $n$, they are real valued for ${\mathcal B} \le 1$. The MD simulations confirm that these constraints put a physical limit during the aggregation phase.

### Granular MD simulations

The equation of motion of the form

56 $$\begin{array}{ccc}\frac{d{{\bf{v}}}_{i}}{dt} & = & \sum _{j}\,[{\rm{\Theta }}({\xi }_{ij})\,({\mathscr{E}}{\xi }_{ij}^{\frac{3}{2}}+{\mathscr{D}}{\xi }_{ij}^{\frac{1}{2}}{\dot{\xi }}_{ij})\,{{\bf{n}}}_{ji}]+{\mathscr{K}}\,\sum _{k}\,\sum _{{\bf{b}}}^{\prime} \frac{{q}_{i}{q}_{k}}{|{{\bf{r}}}_{ki}+{\bf{b}}L{|}^{3}}({{\bf{r}}}_{ki}+{\bf{b}}L),\end{array}$$

is solved for each particle with a setup of periodic boundary conditions in a cubic box of size ${({d}_{0}L)}^{3}=70{d}_{0}\times 70{d}_{0}\times 70{d}_{0}$, where *d*_0_ is the monomer diameter and *L* is the non-dimensional system length [Tables 2 and 3]. Here **b** is a vector of integers representing the periodic replicas of the system in each Cartesian direction. The symbol ′ indicates that $k\ne i$ if ${\bf{b}}=0$ to avoid Coulomb interaction of particles with themselves. The non-dimensional numbers in the above equation are ${\mathcal E} =\frac{\alpha {l}_{{\rm{ref}}}^{\mathrm{3/2}}{t}_{{\rm{ref}}}}{{m}_{{\rm{ref}}}{v}_{{\rm{ref}}}}$, ${\mathscr{D}}=\frac{\beta {l}_{{\rm{ref}}}^{\mathrm{1/2}}{t}_{{\rm{ref}}}}{{m}_{{\rm{ref}}}}$ and ${\mathscr{K}}=\frac{{k}_{e}{q}_{{\rm{ref}}}^{2}{t}_{{\rm{ref}}}}{{m}_{{\rm{ref}}}{v}_{{\rm{ref}}}{l}_{{\rm{ref}}}^{2}}$, with *α* and *β* being viscoelatic material constants. For practicality, we select the reference length ${l}_{{\rm{ref}}}\equiv {d}_{0}$, time reference ${t}_{{\rm{ref}}}$, velocity reference ${v}_{{\rm{ref}}}$, and charge reference ${q}_{{\rm{ref}}}$ such that the elastic force strength ${\mathcal E} \approx 278$, dissipative force strength ${\mathscr{D}}={\mathscr{E}}$/10, and Coulomb force strength is varied across ${\mathscr{K}}=0.4-5.0$ [Tables 2 and 3]. The effect of dissipation compared to elastic forces is extensively studied for neutral systems^27^. We have reported on the variation of Coulomb strength compared to dissipation and elastic forces in^25^. The above order of magnitudes of ${\mathcal E}$, ${\mathscr{D}}$, and ${\mathscr{K}}$ also help to attain an early clustering in non-dimensional time units in a finite size ($N\sim 50000$) neutral granular gas system [see^25^ for more details]. Also ${\xi }_{ij}\equiv {d}_{0}-{r}_{ij}$, ${\dot{\xi }}_{ij}\equiv \frac{d\xi }{dt}$, **n**_*ji*_ is the unit vector pointing from center of in-contact neighbor *j* towards the center of particle *i*, while **r**_*ki*_ is the distance vector pointing from particle *k* towards the center of particle *i*.

**Table 2:** Simulation parameters.

Parameter	Expression	Value
${N}_{{\rm{mon}}}$	No. of monomers	$8516$, $19652$, $50016$, $89746$
${L}^{3}$	System size	$70\times 70\times 70$
$\varphi$	Monomer filling fraction	0.013, 0.030, 0.076, 0.137
${\mathcal E}$	$\frac{\alpha {l}_{{\rm{ref}}}^{\mathrm{3/2}}{t}_{{\rm{ref}}}}{{m}_{{\rm{ref}}}{v}_{{\rm{ref}}}}$	278.0
${\mathscr{D}}$	$\frac{\beta {l}_{{\rm{ref}}}^{\mathrm{1/2}}{t}_{{\rm{ref}}}}{{m}_{{\rm{ref}}}}$	27.8
${\mathscr{K}}$	$\frac{{k}_{e}{q}_{{\rm{ref}}}^{2}{t}_{{\rm{ref}}}}{{m}_{{\rm{ref}}}{v}_{{\rm{ref}}}{l}_{{\rm{ref}}}^{2}}$	0.4, 1.0, 5.0

**Table 3:** Reference values in SI units for conversion of non-dimensional results to laboratory relevant values.

Reference scale	Description	Value
${m}_{{\rm{ref}}}$	Particle mass reference	$1.52\times {10}^{-4}\,{\rm{kg}}$
${l}_{{\rm{ref}}}={d}_{0}$	Length reference=monomer diameter	$4.78\times {10}^{-3}\,{\rm{m}}$
${v}_{{\rm{ref}}}$	Velocity reference	1.0 ms^−1^
${t}_{{\rm{ref}}}={d}_{0}/{v}_{{\rm{ref}}}$	Time reference reference	$4.78\times {10}^{-3}\,{\rm{s}}$
${T}_{{\rm{ref}}}=\frac{1}{3}{m}_{{\rm{ref}}}{v}_{{\rm{ref}}}^{2}$	Temperature reference	$0.51\times {10}^{-4}\,{\rm{kg}}\,{{\rm{m}}}^{2}\,{{\rm{s}}}^{-2}$
*k*_*e*_	Coulomb’s constant	$8.98\times {10}^{9}\,{\rm{N}}\,{{\rm{m}}}^{2}\,{{\rm{C}}}^{-2}$
*α*	Elastic constant of particles	$2.67\times {10}^{4}\,{\rm{kg}}\,{{\rm{m}}}^{-1/2}\,{{\rm{s}}}^{-2}$
*β*	Viscous constant of particles	$1.28\times {10}^{1}\,{\rm{kg}}\,{{\rm{m}}}^{-1/2}\,{{\rm{s}}}^{-1}$
*q*_ref_	Charge reference	$2.0\times {10}^{-8}\,{\rm{C}}$–$5.6\times {10}^{-9}\,{\rm{C}}$

The equation of charge on particle *i* may be written as

57 $$\frac{d{q}_{i}}{dt}=\sum _{j}\,[{\rm{\Theta }}({\xi }_{ij}){I}_{ji}],$$

with *I*_*ji*_ being the charge-exchange currents from colliding neighbors *j* during the course of collision. For any contact neighbor *j*, we approximate its integrated value over the time  interval $\tau$ by Eq. (28), *i*.*e*.

58 $$({q}_{i}^{{\rm{^{\prime} }}}-{q}_{i})=\int \,d{q}_{i}={\int }_{0}^{\tau }\,{I}_{ji}dt\approx {C}_{{\rm{\Delta }}q}|{{\bf{v}}}_{ij}\cdot {\bf{n}}{|}^{\eta }\frac{{q}_{i}-{q}_{0}}{|{q}_{i}-{q}_{0}|}.$$

See^25^ for more details. The long-range Coulomb forces for a setup with periodic boundary conditions in Eq. (56) is challenging and conditionally convergent as it depends on the order of summation. We employ the Ewald summation that converges rapidly, and has a computational complexity ${\mathscr{O}}({N}_{{\rm{m}}{\rm{o}}{\rm{n}}}^{3/2})$. The algorithm is highly parallelized and optimized on graphics processing unit (GPU). In our simulations, the total computing time to reach non-dimensional simulation time ~10^3^ for a typical simulation with monomers ${N}_{{\rm{m}}{\rm{o}}{\rm{n}}}\sim {10}^{5}$, including the long-range electrostatic forces, is of the order of weeks. See^25^ for more details.

### Comparison of individual *T*, 〈*δq*^2^〉, and *n* profiles, and emergence of advective flow using Mach number

**Figure 7:**
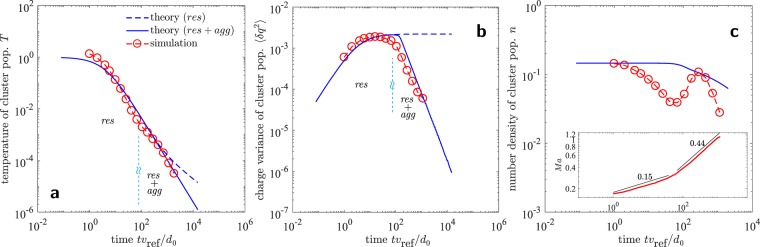
Individual comparisons of (**a**) temperature of cluster population $T$, (**b**) charge variance of cluster population $\langle \delta {q}^{2}\rangle$, and (**c**) number density of cluster population $n$, with the theoretical predictions. The granular temperature, charge variance and average size of the cluster population during aggregation evolve in such a manner that their non-dimensional combination ${\mathcal B} (t)={k}_{e}\langle \delta {q}^{2}\rangle /(Td)\le 1$ (main text). The number density evolution, however, is highly dynamic and exhibits a non-monotonic behavior due to emergence of mesoscopic flow, marked by a transition in the rate of increase of Mach number *Ma*, shown in (**c**) inset. The numbers in (**c**) inset are the power law exponents of the time dependence of *Ma*. The short-hands *res* and *agg* denote restitution and aggregation respectively.

In Fig. 7, we decompose the theoretical comparison of ${\mathcal B}$, presented in the main text, into individual comparisons of $T$, $\langle \delta {q}^{2}\rangle$, and $n$ profiles for a typical simulation run. The difference between the kinetic theory with and without aggregation is also emphasized.

It is noticeable that the granular temperature of the aggregates closely follows Haff’s law, and is confirmed by theory, notwithstanding the presence of long-range effects and intricate aggregation and annihilation events. If only the restitutive terms of the hydrodynamic equations are considered (dashed line), the theory predicts that $T$ drops at a slower rate at long times. Furthermore, the charge variance in this case saturates. The number density in the absence of aggregation is, of course, invariant. If the aggregation dynamics is augmented, the simulation results are closely predicted by the theory.

In the MD simulations, the decay of $\langle \delta {q}^{2}\rangle$ during the aggregation phase closely agrees with the theory, even though we observe that the charge exchange in the simulations leads to a symmetric but non-Gaussian charge distribution among monomers during the initial restitution phase [Fig. 1].

The number density evolution, however, is highly dynamic and exhibits a non-monotonic behavior due to fragmentation events caused by the emergence of mesoscopic flow (see Suppl. Movie). The theory predicts the decay of cluster density only in an average sense [Fig. 7(c) inset]. To quantify the emergence of the mesoscopic flow, we calculate the Mach number

59 $$Ma=\frac{{\langle {{\bf{V}}}^{2}\rangle }^{1/2}}{{v}_{{\rm{t}}{\rm{h}}}},$$

where **V** is the local mesoscopic velocity, and ${v}_{{\rm{t}}{\rm{h}}}\sim \sqrt{T}$ is the thermal velocity. To compute this, we divide the system volume into equal sized cubic boxes. The advective velocity in *j*^*th*^ box is computed as ${{\bf{V}}}_{j}={\sum }_{i}\,{m}_{i}{{\bf{v}}}_{i}/{\sum }_{i}\,{m}_{i}$, where index *i* runs over all the monomers in that box *j*. We take the square of this velocity (we square the velocity of the box before summing it up over all the boxes, otherwise the advective velocities of the boxes might cancel each other even in the presence of a directed motion/advective flow, for example in a lattice of vortices) as $\langle {{\bf{V}}}^{2}\rangle =\frac{1}{{N}_{{\rm{boxes}}}}\,{\sum }_{j}\,{{\bf{V}}}_{j}^{2}$, take its square root, and normalize it with the thermal velocity ${v}_{{\rm{t}}{\rm{h}}}\sim \sqrt{T}$. Thus according to our definition, *Ma* is a global measure of the magnitude of directed/advective motion. The time evolution of *Ma* is shown in Fig. 7(c) (inset), which indicates that *Ma* grows at a higher rate when the number density evolution becomes non-monotonic ($t{v}_{{\rm{ref}}}/{d}_{0}\approx {10}^{2}$), indicating an intricate aggregation and fragmentation dynamics, and the generation of mesoscopic flow.

### Reference scales and laboratory relevance of present results

**Figure 8:**
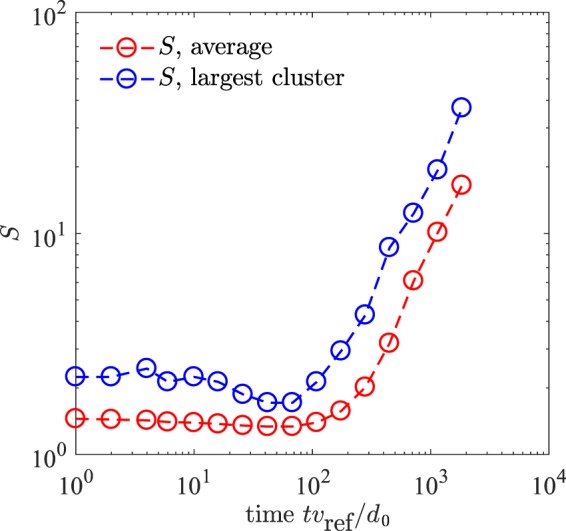
Growth of the average cluster size, and the size of the largest cluster.

Typically non-Brownian growth in planetary dust becomes dominant for monomer sizes near or above several *μm*^46^ and the growth barrier problem^12^ starts to arise near $d\sim {10}^{-3}\,{\rm{m}}$. The mass of silica particles in this range of sizes is $m\sim {10}^{-4}-{10}^{-6}\,{\rm{kg}}$. If the particles are initially agitated with velocities $v\sim 1.0\,{\rm{m}}\,{{\rm{s}}}^{-1}$, the time scale reference to convert our simulation time to laboratory time is $d/{v}_{{\rm{ref}}}\sim 4.78\times {10}^{-3}\,{\rm{s}}$. Thus in our results the growth over 10^4^ units of non-dimensional time approximately implies growth over ~10 s. The average size of aggregates in the growth period grows approximately by one order of magnitude (*e*.*g*. the growth of the largest cluster is from ≈2 mm to ≈7 cm in ≈10 s time for particles of such size and mass, and for initial monomer filling fraction of $\varphi =0.076$) [Fig. 8].

### Statistical and analytical methods

The following method codes are used:

1. An in-house MATLAB program to solve Eqs (6–8).
2. An in-house cluster analysis code in MATLAB to obtain the fractal dimension, cluster size distribution, average cluster size, and other statistical quantities, are made available at https://gitlab.com/cphyme/cluster-analysis.

## Supplementary information

Mathematica instructions to solve kinetic integrals

Movie of aggregating charged granular gas
