# Supplementary material for: Electrification in granular gases leads to constrained fractal growth
Source: Sci Rep. 2019 Jun 21;9:9049. doi: 10.1038/s41598-019-45447-x (PMC6588598; doi:10.1038/s41598-019-45447-x)
Supplement: Supplementary file 1 — Mathematica instructions to solve kinetic integrals [file 41598_2019_45447_MOESM1_ESM.pdf]

Supplementary information: Electrification in granular gases leads to constrained fractal growth

Chamkor Singh[1,2] **and Marco G. Mazza**[1,3,*]

[1]Max Planck Institute for Dynamics and Self-Organization (MPIDS), 37077, Göttingen, Germany
[2]Georg-August-Universität Göttingen, Friedrich-Hund-Platz 1, 37077 Göttingen, Germany
[3]Interdisciplinary Centre for Mathematical Modelling and Department of Mathematical Sciences, Loughborough University, Loughborough, Leicestershire LE11 3TU, United Kingdom
[*]marco.mazza@ds.mpg.de

```
(*This is the MATHEMATICA instructions
 to solve the restitutive and aggregative integrals
  Some of the integrals below are time expensive
 and might take long times in MATHEMATICA.
  The last instruction at the bottom of this
 script can be used to clear all the results,
  so that the script can be run again.*)

Integrate[x^2 / (E^(b x y) E^x^2 E^y^2), {y, 0, Infinity}, {x, 0, Infinity}]

Integrate[x^2 / (E^(b x y) E^x^2 E^y^2), {y, -Infinity, 0}, {x, -Infinity, 0}]

Integrate[x^2 / (E^(b x y) E^x^2 E^y^2), {y, 0, Infinity}, {x, 0, Infinity}] +
 Integrate[x^2 / (E^(b x y) E^x^2 E^y^2), {y, -Infinity, 0}, {x, -Infinity, 0}]
```

$$\text{ConditionalExpression}\left[\frac{b}{2\left(-4+b^2\right)}-\frac{b\sqrt{4-b^2}-2\pi+4\,\text{ArcTan}\left[\frac{b}{\sqrt{4-b^2}}\right]}{2\left(4-b^2\right)^{3/2}}+\frac{2\,\text{ArcTan}\left[\frac{\sqrt{4-b^2}}{b}\right]}{\left(4-b^2\right)^{3/2}},\ \text{Re}\left[b^2\right]<4\,\&\&\,\text{Re}[b]>0\right]$$

$$\text{Simplify}\left[\frac{b}{2\left(-4+b^2\right)}-\frac{b\sqrt{4-b^2}-2\pi+4\,\text{ArcTan}\left[\frac{b}{\sqrt{4-b^2}}\right]}{2\left(4-b^2\right)^{3/2}}+\frac{2\,\text{ArcTan}\left[\frac{\sqrt{4-b^2}}{b}\right]}{\left(4-b^2\right)^{3/2}}\right]$$

$$\frac{-b\sqrt{4-b^2}+\pi-2\,\text{ArcTan}\left[\frac{b}{\sqrt{4-b^2}}\right]+2\,\text{ArcTan}\left[\frac{\sqrt{4-b^2}}{b}\right]}{\left(4-b^2\right)^{3/2}}$$

$$\text{Simplify}\left[\frac{-b\sqrt{4-b^2}+\pi-2\,\text{ArcTan}\left[\frac{b}{\sqrt{4-b^2}}\right]+2\,\text{ArcTan}\left[\frac{\sqrt{4-b^2}}{b}\right]}{\left(4-b^2\right)^{3/2}}\right]$$

```
Integrate[E^(-x^2 - y^2) x^2 (1 - b x y), {y, 0, Infinity}, {x, -Infinity, 0}]

Integrate[E^(-x^2 - y^2) x^2 (1 - b x y), {y, 0, Infinity}, {x, -Infinity, 0}] +
 Integrate[E^(-x^2 - y^2) x^2 (1 - b x y), {y, -Infinity, 0}, {x, 0, Infinity}]
```

```
Integrate[r (2 + Abs[b] r^2 Sin[t] Cos[t]) E^(-Abs[b] r^2 Sin[t] Cos[t]) E^(-r^2),
  {r, 0, Infinity}, {t, 0, Pi / 2}] +
 Integrate[r (2 + Abs[b] r^2 Sin[t] Cos[t]) E^(-Abs[b] r^2 Sin[t] Cos[t]) E^(-r^2),
  {r, 0, Infinity}, {t, Pi, 3 Pi / 2}]
```

```
Integrate[E^(-x^2 - y^2 - b x y) (2 + b x y),
 {y, -Infinity, Infinity}, {x, -Infinity, Infinity}]
```

```
Integrate[x^2 / (E^(b x y) E^x^2 E^y^2),
 {y, -Infinity, Infinity}, {x, -Infinity, Infinity}]
```

```
Integrate[E^(-x^2) E^(-y^2) E^(-R Abs[x y])
  (2 + 2 R Abs[x y] + R^2 (Abs[x y])^2 - R x y - R^2 Abs[x y] x y),
 {y, -Infinity, Infinity}, {x, -Infinity, Infinity}]
```

```
Integrate[x^2 E^(-x^2) E^(-y^2) E^(-R Abs[x y]) (-R x y + R Abs[x y] + 1 ),
 {y, -Infinity, Infinity}, {x, -Infinity, Infinity}]
```

```
Integrate[E^(-x^2) E^(-y^2) E^(-R Abs[x y])
   (-R x y E^(R Abs[x y]) + R x y + E^(R Abs[x y]) - R Abs[x y] - 1 ),
  {y, -Infinity, 0}, {x, 0, Infinity}] + Integrate[E^(-x^2) E^(-y^2)
   E^(-R Abs[x y]) (-R x y E^(R Abs[x y]) + R x y + E^(R Abs[x y]) - R Abs[x y] - 1 ),
  {y, 0, Infinity}, {x, -Infinity, 0}]
```

```
Integrate[E^(-x^2) E^(-y^2) E^(-R Abs[x y])
  ( P A x y (1 + R Abs[x y]) + B (2 + 2 R Abs[x y] + R^2 (Abs[x y])^2) ),
 {y, -Infinity, Infinity}, {x, -Infinity, Infinity}]
```

```
Integrate[
  E^(-x^2) E^(-y^2) E^(-R Abs[x y]) (x y Q (-R Abs[x y] + E^(R Abs[x y]) - 1) +
     R^2 (Abs[x y])^2 + 2 R Abs[x y] - 2 E^(R Abs[x y]) + 2), {y, -Infinity, 0},
  {x, 0, Infinity}] + Integrate[E^(-x^2) E^(-y^2) E^(-R Abs[x y])
   (x y Q (-R Abs[x y] + E^(R Abs[x y]) - 1) + R^2 (Abs[x y])^2 +
     2 R Abs[x y] - 2 E^(R Abs[x y]) + 2), {y, 0, Infinity}, {x, -Infinity, 0}]
```

```
Integrate[(w^5 (-B w^(16 / 5)) (1 - a / w^2)) / E^w^2, {w, Sqrt[b], Infinity}]
```

```
Integrate[
  E^(-x^2) E^(-y^2) E^(-R Abs[x y]) (R x y (- R Abs[x y] + E^(R Abs[x y]) - 1 ) +
     (R Abs[x y])^2 + 2 R Abs[x y] - 2 E^(R Abs[x y]) + 2 ) , {y, -Infinity, 0},
  {x, 0, Infinity}] + Integrate[E^(-x^2) E^(-y^2) E^(-R Abs[x y])
   (R x y (- R Abs[x y] + E^(R Abs[x y]) - 1 ) + (R Abs[x y])^2 + 2 R Abs[x y] -
     2 E^(R Abs[x y]) + 2 ) , {y, 0, Infinity}, {x, -Infinity, 0}]
```

```
Integrate[
 Sin[x] (A (Abs[Cos[x]])^(2 * k + 1) + B (Abs[Cos[x]])^(k + 1)), {x, Pi / 2, Pi}]
```

```
Integrate[w^2 E^(-w^2) (1 - a / w^2) (A w^(2 * k + 1) + B w^(k + 1)),
 {w, Sqrt[b], Infinity}]
```

```
Integrate[
 E^(-x^2) E^(-y^2) E^(-R Abs[x y]) ( (1 + R Abs[x y]) (R x y) A + S Abs[x - y] R x y B -
    2 (1 + R Abs[x y] + (R Abs[x y])^2 ) A / 2 - S Abs[x - y] (1 + R Abs[x y]) B ),
 {y, -Infinity, Infinity}, {x, -Infinity, Infinity}]
```

```
Integrate[(w^(26 / 5) (1 - Tanh[1 / w^2])) / E^w^2, {w, sqrt[1], Infinity}]
```

```
Integrate[E^(-x^2) E^(-y^2) E^(-R Abs[x y])
   (-R x y E^(-R Abs[x y]) + R x y + E^(R Abs[x y]) - R Abs[x y] - 1 ) ,
  {y, -Infinity, 0}, {x, 0, Infinity}] + Integrate[E^(-x^2) E^(-y^2)
   E^(-R Abs[x y]) (-R x y E^(-R Abs[x y]) + R x y + E^(R Abs[x y]) - R Abs[x y] - 1 ),
  {y, 0, Infinity}, {x, -Infinity, 0}]
```

```
Integrate[x y E^(-x^2) E^(-y^2) E^(-R Abs[x y])
   (-R x y E^(-R Abs[x y]) + R x y + E^(R Abs[x y]) - R Abs[x y] - 1 ) ,
  {y, -Infinity, 0}, {x, 0, Infinity}] + Integrate[x y E^(-x^2) E^(-y^2)
   E^(-R Abs[x y]) (-R x y E^(-R Abs[x y]) + R x y + E^(R Abs[x y]) - R Abs[x y] - 1 ),
  {y, 0, Infinity}, {x, -Infinity, 0}]
```

```
Integrate[(b^2) / (1 + a b^2), {b, 0, c}]
```

```
Integrate[((p x^2 y^2 - (x^2 y^2 / a)^(3 / 2) (b / (x y))) (1 / (x y))) / E^(x^2 / s),
 {x, -Infinity, Infinity}]
```

```
Integrate[b / (1 + a b^2), {b, 0, c}]
```

```
Integrate[(x^2 Log[r / x^2]) / E^(x^2 / s), {x, -Infinity, Infinity}]
```

```
(*Execute following to clear all notebook inputs*)
NotebookDelete[Cells[EvaluationNotebook[], GeneratedCell → True]]
```
